# Long-term neuropsychological trajectories in children with epilepsy: does surgery halt decline?

**DOI:** 10.1093/brain/awae121

**Published:** 2024-04-20

**Authors:** Maria H Eriksson, Freya Prentice, Rory J Piper, Konrad Wagstyl, Sophie Adler, Aswin Chari, John Booth, Friederike Moeller, Krishna Das, Christin Eltze, Gerald Cooray, Ana Perez Caballero, Lara Menzies, Amy McTague, Sara Shavel-Jessop, Martin M Tisdall, J Helen Cross, Patricia Martin Sanfilippo, Torsten Baldeweg

**Affiliations:** Developmental Neurosciences Research and Teaching Department, UCL Great Ormond Street Institute of Child Health, London, WC1N 1EH, UK; Department of Neuropsychology, Great Ormond Street Hospital for Children, London, WC1N 3JH, UK; Department of Neurology, Great Ormond Street Hospital for Children, London, WC1N 3JH, UK; Developmental Neurosciences Research and Teaching Department, UCL Great Ormond Street Institute of Child Health, London, WC1N 1EH, UK; Department of Neuropsychology, Great Ormond Street Hospital for Children, London, WC1N 3JH, UK; Developmental Neurosciences Research and Teaching Department, UCL Great Ormond Street Institute of Child Health, London, WC1N 1EH, UK; Department of Neurosurgery, Great Ormond Street Hospital for Children, London, WC1N 3JH, UK; Department of Imaging Neuroscience, UCL Queen Square Institute of Neurology, London, WC1N 3BG, UK; Developmental Neurosciences Research and Teaching Department, UCL Great Ormond Street Institute of Child Health, London, WC1N 1EH, UK; Developmental Neurosciences Research and Teaching Department, UCL Great Ormond Street Institute of Child Health, London, WC1N 1EH, UK; Department of Neurosurgery, Great Ormond Street Hospital for Children, London, WC1N 3JH, UK; Data Research, Innovation and Virtual Environments Unit, Great Ormond Street Hospital for Children, London, WC1N 3JH, UK; Department of Neurophysiology, Great Ormond Street Hospital for Children, London, WC1N 3JH, UK; Department of Neurology, Great Ormond Street Hospital for Children, London, WC1N 3JH, UK; Department of Neurophysiology, Great Ormond Street Hospital for Children, London, WC1N 3JH, UK; Department of Neurophysiology, Great Ormond Street Hospital for Children, London, WC1N 3JH, UK; Department of Neurophysiology, Great Ormond Street Hospital for Children, London, WC1N 3JH, UK; Department of Clinical Neuroscience, Karolinska Institutet, Solna 171 77, Sweden; North Thames Genomic Laboratory Hub, Great Ormond Street Hospital for Children, London, WC1N 3JH, UK; Department of Clinical Genetics, Great Ormond Street Hospital for Children, London, WC1N 3JH, UK; Developmental Neurosciences Research and Teaching Department, UCL Great Ormond Street Institute of Child Health, London, WC1N 1EH, UK; Department of Clinical Genetics, Great Ormond Street Hospital for Children, London, WC1N 3JH, UK; Developmental Neurosciences Research and Teaching Department, UCL Great Ormond Street Institute of Child Health, London, WC1N 1EH, UK; Department of Neuropsychology, Great Ormond Street Hospital for Children, London, WC1N 3JH, UK; Developmental Neurosciences Research and Teaching Department, UCL Great Ormond Street Institute of Child Health, London, WC1N 1EH, UK; Department of Neurosurgery, Great Ormond Street Hospital for Children, London, WC1N 3JH, UK; Developmental Neurosciences Research and Teaching Department, UCL Great Ormond Street Institute of Child Health, London, WC1N 1EH, UK; Department of Neurology, Great Ormond Street Hospital for Children, London, WC1N 3JH, UK; Department of Neurosurgery, Great Ormond Street Hospital for Children, London, WC1N 3JH, UK; Young Epilepsy, Lingfield, RH7 6PW, UK; Developmental Neurosciences Research and Teaching Department, UCL Great Ormond Street Institute of Child Health, London, WC1N 1EH, UK; Department of Neuropsychology, Great Ormond Street Hospital for Children, London, WC1N 3JH, UK; Developmental Neurosciences Research and Teaching Department, UCL Great Ormond Street Institute of Child Health, London, WC1N 1EH, UK; Department of Neuropsychology, Great Ormond Street Hospital for Children, London, WC1N 3JH, UK; Department of Neurosurgery, Great Ormond Street Hospital for Children, London, WC1N 3JH, UK

**Keywords:** epilepsy surgery, paediatric, neuropsychology, IQ, academic attainment, trajectories

## Abstract

Neuropsychological impairments are common in children with drug-resistant epilepsy. It has been proposed that epilepsy surgery might alleviate these impairments by providing seizure freedom; however, findings from prior studies have been inconsistent. We mapped long-term neuropsychological trajectories in children before and after undergoing epilepsy surgery, to measure the impact of disease course and surgery on functioning.

We performed a retrospective cohort study of 882 children who had undergone epilepsy surgery at Great Ormond Street Hospital (1990–2018). We extracted patient information and neuropsychological functioning [obtained from IQ tests (domains: full-scale IQ, verbal IQ, performance IQ, working memory and processing speed) and tests of academic attainment (reading, spelling and numeracy)] and investigated changes in functioning using regression analyses.

We identified 500 children (248 females) who had undergone epilepsy surgery [median age at surgery = 11.9 years, interquartile range = (7.8, 15.0)] and neuropsychological assessment. These children showed declines in all domains of neuropsychological functioning in the time leading up to surgery (all *P*-values ≤0.001; e.g. β_FSIQ_ = −1.9, SE_FSIQ_ = 0.3, *P*_FSIQ_ < 0.001). Children lost on average one to four points per year, depending on the domain considered; 27%–43% declined by ≥10 points from their first to their last preoperative assessment. At the time of presurgical evaluation, most children (46%–60%) scored one or more standard deviations below the mean (<85) on the different neuropsychological domains; 37% of these met the threshold for intellectual disability (full-scale IQ < 70). On a group level, there was no change in performance from pre- to postoperative assessment on any of the domains (all *P*-values ≥0.128). However, children who became seizure free through surgery showed higher postoperative neuropsychological performance (e.g. *r*_rb-FSIQ_ = 0.37, *P* < 0.001). These children continued to demonstrate improvements in neuropsychological functioning over the course of their long-term follow-up (e.g. β_FSIQ_ = 0.9, SE_FSIQ_ = 0.3, *P*_FSIQ_ = 0.004). Children who had discontinued antiseizure medication treatment at 1-year follow-up showed an 8- to 13-point advantage in postoperative working memory, processing speed and numeracy, and greater improvements in verbal IQ, working memory, reading and spelling (all *P*-values ≤0.034) over the postoperative period compared with children who were seizure free and still receiving antiseizure medication.

In conclusion, by providing seizure freedom and the opportunity for antiseizure medication cessation, epilepsy surgery might not only halt but reverse the downward trajectory that children with drug-resistant epilepsy display in neuropsychological functioning. To halt this decline as soon as possible or, potentially, to prevent it from occurring in the first place, children with focal epilepsy should be considered for epilepsy surgery as early as possible after diagnosis.

## Teaser

In a retrospective cohort study, Eriksson *et al.* examined neuropsychological trajectories in children who underwent surgery for drug-resistant epilepsy. In most children, surgery was able to halt and even reverse a downward trajectory in neuropsychological functioning by providing seizure freedom and the opportunity to discontinue antiseizure medication.

## Introduction

Children with epilepsy display high rates of neuropsychological impairment,^1^ which can significantly impact their academic attainment, social relationships and eventual employment opportunities.^2^ They may present these difficulties prior to the onset of their epilepsy or develop them over the course of their epilepsy.^3^ A range of factors have been attributed as causes of these impairments, including underlying aetiology, ongoing seizures and use of antiseizure medication (ASM).^4^

Epilepsy surgery is a low-risk and widely accepted form of treatment for carefully selected children with drug-resistant epilepsy.^5^ Approximately 67% of children are seizure free 1 year after surgery,^6^ and 60% of children maintain their seizure freedom 5 years after surgery.^7^ Although the primary aim of epilepsy surgery is to provide seizure freedom, there exists an underlying belief or hope that it might also have a beneficial impact on cognition. Still, the documented effects of surgery on cognition in children with epilepsy remain inconsistent.^5^ Some studies have reported improvements in performance from pre- to postoperative neuropsychology assessment,^8-26^ whereas others have found no change^8,9,11,12,14,19,20,22,23,25,27-44^ or declines^21,22,25,27,28,30,42^ across different domains of functioning.

In an effort to disentangle these inconsistent findings, researchers have sought to identify determinants of postoperative neuropsychological outcomes.^45-47^ Some of these determinants have demonstrated largely consistent findings: older age of epilepsy onset,^12,14,25,26,48^ fewer ASMs trialled prior to surgery,^49^ higher preoperative neuropsychological scores,^18,20,22,24-28,35,38,40,48,49^ unilobar surgery (as opposed to multilobar surgery^48^ or hemispherotomy^49^), a diagnosis of tumour (as opposed to vascular aetiological causes^49^ or focal cortical dysplasia^38^) and postoperative reduction or withdrawal of ASMs^17,21,40,49^ have been found to be predictive of better postoperative neuropsychological functioning or an improvement from pre- to postoperative neuropsychological assessment. Right-sided surgery has, furthermore, been found to benefit verbal neuropsychological abilities, and left-sided surgery has been found to benefit visuospatial abilities.^9,17,18,39^ Other determinants of neuropsychological outcomes have produced less consistent findings. For example, some studies have shown that younger age at surgery^12,24^ and shorter duration of epilepsy^10,23,26,28,43^ are associated with better neuropsychological outcomes. Other studies have, however, demonstrated the opposite effect for age at surgery^40,48,49^ or failed to find any effect of duration of epilepsy on neuropsychological outcomes.^14,17,49^ Studies that have investigated the impact of postoperative seizure outcome have also had conflicting findings, with some studies demonstrating a beneficial impact of seizure freedom on neuropsychological outcomes^18,20-22,37,39,40,50^ but others finding no effect.^9,10,14,17,24,26,33,35,48,49^ These factors are, however, of particular importance because they are potentially modifiable, informing not only if but also when children should proceed to surgery.

Some of the variability in these findings might be explained by limitations of the studies conducted to date. The majority of studies that have investigated the effect of surgery on neuropsychological outcomes in paediatric epilepsy surgery patients have lacked sufficiently large cohorts, been composed solely of patients with temporal lobe epilepsy, and focused on the effects of surgery on language and/or memory.^51^ Perhaps more importantly, most studies have evaluated the effect of surgery on neuropsychological functioning by measuring change from a single preoperative assessment to that of a single postoperative assessment, typically performed 1 year after surgery. This approach fails to take into account an individual’s preoperative neuropsychological trajectory and changes that might occur later than 1 year after surgery.

There is an assumption that children with drug-resistant epilepsy increasingly fall behind their typically developing peers, giving rise to a downward neuropsychological trajectory.^45^ This is because neuropsychological impairments are common at the time of presurgical evaluation.^52^ However, to the best of our knowledge, only one study to date has described longitudinal changes in neuropsychological performance prior to surgical intervention.^50^ Laguitton *et al*.^50^ found that, in 29 children who had undergone two preoperative assessments (one completed 2–3 years before surgery and another 1 year before surgery) approximately half of the children (55%) declined between assessments.

There is also a lack of studies that have investigated long-term longitudinal changes in neuropsychological functioning after epilepsy surgery. The majority of studies that have included multiple postoperative neuropsychological assessments have examined relatively immediate changes in functioning, typically within 1 year,^20,28,39^ 2 years^15,27,29,33^ or 3 years^10^ of surgery. Studies^17,22,50^ that have reported longitudinal changes ≥4 years after surgery have had limited sample sizes (*n* ≤ 61) and demonstrated mixed findings regarding the impact of postoperative seizure freedom on the potential improvement in neuropsychological functioning. Such long-term trajectories are, however, particularly relevant in children undergoing epilepsy surgery, where the maturation and greater plasticity of the developing brain might allow for the reorganization, and thereby recovery, of brain functions.^53^ It is therefore still a challenge for clinicians to inform caregivers of what they can expect in terms of their child’s cognitive development, not only in the short term but, crucially, in the long term.

To gain a better understanding of the impact of disease course and surgery on neuropsychological functioning in children with drug-resistant focal epilepsy, and thereby improve both clinical decision-making and patient counselling, we studied a large, heterogeneous cohort of children undergoing epilepsy surgery. We aimed to investigate long-term neuropsychological trajectories, both before and after epilepsy surgery, and to identify determinants of these trajectories.

## Materials and methods

### Patient cohort

We included children who had undergone surgical resection or disconnection for epilepsy at Great Ormond Street Hospital (GOSH; London, UK) between 1 January 1990 and 31 December 2018 and who had either a pre- or postoperative neuropsychological assessment available. We excluded children who had undergone surgical neuromodulation (i.e. vagus nerve stimulation, deep brain stimulation or responsive neurostimulation), thermocoagulation or laser interstitial thermal therapy. We also excluded children who had undergone multiple subpial transection because this procedure is no longer routinely performed in children with epilepsy. If children had undergone multiple surgeries over the course of the study period, we included outcomes after their first surgery only.

### Dataset description

We extracted the following information from medical records: patient demographics, handedness, educational status, family history of epilepsy, epilepsy characteristics (including both historical seizures and seizures at time of presurgical evaluation), preoperative MRI findings, preoperative ASMs (both the total number of different ASMs that the patient had trialled from the time of their epilepsy onset to the time of their preoperative evaluation and the number of ASMs that the patient was receiving at the time of their preoperative evaluation), details of their surgery, genetic results, histopathology diagnoses and postoperative outcomes.

We considered patients to be either seizure free (including freedom from auras) or not seizure free 1 year after surgery and noted whether patients were receiving, weaning or no longer receiving ASMs at 1-year follow-up. In patients with additional, long-term postoperative neuropsychological assessments, we also recorded their seizure outcome and postoperative ASM status at final assessment.

### Neuropsychological testing

Neuropsychology assessments are regularly performed preoperatively and 1 year after surgery at GOSH, in line with International League Against Epilepsy (ILAE) guidelines.^54^ Children may, however, undergo multiple pre- and/or postoperative neuropsychological assessments over the course of their epilepsy. The reasons for this (other than epilepsy surgery evaluation) may be caregiver concerns about cognitive development, applications for educational services to support children within mainstream school or to allow them to access special educational needs schools, and follow-up as their epilepsy evolves.

We chose to include every neuropsychological assessment that a child had undergone, to map their individual neuropsychological trajectories both before and after surgical intervention. These assessments had been performed using a variety of tests and test versions, owing to the long study time frame and the wide age range of the children included. Patients might have been tested using different tests over the course of their treatment, and the same patient might have been assessed using two different test versions at different time points. The tests and test versions administered are, however, highly correlated, as documented in their technical manuals. Furthermore, the tests have demonstrated robust test–retest reliability. For example, the technical manual of the Wechsler Intelligence Scale for Children—Fourth Edition (WISC-IV) includes test–retest coefficients of 0.86–0.93, depending on the subdomain considered.^55^ The WISC-IV has also demonstrated suitable test–retest reliability specifically in children with epilepsy.^56^

#### Test domains and versions

We included cognitive assessments that had been performed using the WISC (versions WISC-III to WISC-V and the WISC-Revised), the Wechsler Preschool and Primary Scale of Intelligence (WPPSI; versions WPPSI-III, WPPSI-IV and WPPSI-Revised), the Wechsler Adult Intelligence Scale (WAIS; versions WAIS-III, WAIS-IV and WAIS-Revised) and the Wechsler Abbreviated Scale of Intelligence (WASI).

We also included assessments of academic attainment that had been performed using the Wechsler Individual Achievement Test (WIAT; versions WIAT-II and WIAT-III), the Wechsler Objective Reading Dimensions (WORD), the Wechsler Objective Numerical Dimensions (WOND) and the Wide Range Achievement Test (WRAT; version WRAT-III).

We did not include tests developed specifically to assess language and/or memory [e.g. the Clinical Evaluation of Language Fundamentals (CELF)] because in the past these were not consistently administered to children undergoing epilepsy surgery, but rather exclusively to children with existing language and/or memory problems or children who were being evaluated primarily for temporal lobe surgery or hemispherotomy. We did not include developmental tests that can be used to generate developmental quotient (DQ) scores [as opposed to intelligence quotient (IQ) scores]. Such tests, including the Bayley Scales of Infant and Toddler Development, the Mullen Scales of Early Learning and the Griffiths Scales of Child Development, are typically used when a child cannot access an IQ test. This could be because of their chronological age (i.e. infants who are too young to access an IQ test) or developmental age (i.e. children who present with atypical development or developmental delay). The reason we chose to not include developmental tests is because these focus on skills and functions (e.g. reaching and grasping), which arguably cannot be equated with what IQ tests seek to conceptualize (i.e. general cognitive functioning). The convergent validity of developmental and IQ tests is therefore questionable. Indeed, it has been demonstrated that developmental tests are poor predictors of later IQ.^57,58^ It has also been demonstrated that there are differential determinants of IQ and DQ scores in children undergoing epilepsy surgery.^59^

We extracted standard scores for five domains of cognition: full-scale IQ (FSIQ), verbal IQ, performance IQ, working memory and processing speed. Supplementary Table 1 shows how the different indices from the various cognitive tests loaded onto each of these domains. We also extracted standard scores for three domains of academic attainment: reading, spelling and numeracy.

#### Classifying pre- and postoperative performance

We considered the assessments performed closest to surgery as the ‘preoperative’ and ‘postoperative’ neuropsychology assessments of the patients.

We categorized the preoperative neuropsychological performance of the patients into one of four categories: ‘<70’ (two standard deviations or more below the mean; clinical threshold for intellectual disability^60^); ‘70–85’ (within one standard deviation below the mean); ‘85–115’ (within average range); and ‘>115’ (above average).

#### Change from pre- to postoperative performance

In accordance with past studies,^45,50,59^ we considered patients who had an increase or decrease of ≥10 points between their pre- and postoperative neuropsychological assessments to have experienced an improvement or decline in functioning, respectively. Any potential decline discussed hereinafter represents a decline in standard (not raw) scores. A decline in standard scores could reflect a delayed development in comparison to typically developing children, a plateau in development or a loss of skills.

### Statistical analysis

We calculated the descriptive statistics for the cohort using mean (M) with standard deviation (SD), median (Md) with interquartile range (IQR) and count with proportion, as appropriate.

We visualized the effect of duration of epilepsy on preoperative neuropsychological performance both cross-sectionally and longitudinally. For the cross-sectional visualizations, only a single assessment for each patient was included. For the longitudinal visualizations, only patients with two or more assessments were included, and every assessment was included. We fitted linear and generalized additive models to the visualized data-points.

We checked whether the continuous data were normally distributed using Shapiro–Wilk tests.^61^ If the data were normally distributed, we investigated changes in performance from pre- to postoperative performance using dependent-samples *t*-tests. If the data were not normally distributed, we used Wilcoxon signed-rank tests. We further investigated associations between demographic, clinical and surgical variables and neuropsychological functioning using Pearson’s correlation coefficient, Spearman’s rank correlation coefficient and rank-biserial correlation coefficient, as appropriate. We analysed age of epilepsy onset, age at surgery and duration of epilepsy separately, using partial correlations, to take into account the intercorrelated nature between these clinical characteristics.

We performed a series of mixed-effects linear regression analyses to investigate potential moderators of pre- and postoperative neuropsychological trajectories. We chose a mixed-effects model approach because it allows for the inclusion of multiple data-points produced by the same individual, as in the case of patients who have multiple pre- and/or postoperative assessments. To confirm that a mixed-effects structure was justified, we first compared the Akaike information criterion (AIC) of the baseline (fixed effects) model with the AIC of the mixed-effects model (with random intercepts). We then compared the AIC of the mixed-effects model with random intercepts to the AIC of the mixed-effects model with both random intercepts and random slopes, to determine which model fitted the data better. To investigate whether patients showed a decline in neuropsychological functioning prior to surgery, we first tested whether there was a main effect of duration of epilepsy on preoperative neuropsychological functioning. We then investigated whether there was an interaction effect between duration of epilepsy and aetiology on preoperative neuropsychological functioning. We chose Rasmussen encephalitis as the reference category, owing to its known progressive cognitive deterioration.^62^ We compared the AICs of the two mixed-effect models and ran a chi-square test, to choose the model that fitted the data best (significant *P*-value and lower AIC). We repeated this procedure on the postoperative data, with a main effect of time since surgery and with an interaction effect between time since surgery and seizure outcome, to investigate whether seizure freedom impacted upon the potential of patients to improve in neuropsychological functioning postoperatively.

We investigated whether there were differences in demographic and clinical characteristics between patients with a single versus multiple pre- and/or postoperative neuropsychological assessments using chi-square tests for categorical variables and Spearman’s rank-order correlation for skewed continuous variables. Likewise, we investigated whether there were differences in demographic and clinical characteristics between patients who were seizure free after surgery and patients who were not seizure free after surgery. We also checked for differences in demographic and clinical characteristics between patients who had undergone neuropsychological assessment (and were therefore included in the present study) and patients who had had epilepsy surgery but had not undergone neuropsychological assessment (and were therefore excluded from the present study). We performed a series of two-way repeated ANOVA tests to investigate whether there was an interaction effect between time point of preoperative assessment (first versus last preoperative assessment) and seizure outcome on preoperative neuropsychological functioning. Finally, we investigated differences in both postoperative performance and postoperative trajectories in seizure-free patients who had been weaned off ASMs versus those who were still receiving or weaning ASMs using dependent-samples *t*-tests. If the data were not normally distributed, we used Wilcoxon signed-rank tests.

We performed all statistical analyses and visualizations in R v.3.6.3. All tests were two-tailed, and we set the threshold for significance *a priori* at *P* < 0.05. We corrected for multiple comparisons where appropriate using the Holm method^63^ and reported both original and corrected *P*-values.

## Results

### Demographic information and clinical characteristics

From a total of 882 children who had undergone an epilepsy surgery within our inclusion criteria, we identified 500 children who had at least one pre- or postoperative neuropsychological assessment. An overview of available assessments is demonstrated in Supplementary Table 2.

Demographic information and clinical characteristics for the included patients are provided in Supplementary Table 3. Seizure information, including historical seizures and seizures at the time of presurgical evaluation, are reported in Supplementary Table 4. There was an even distribution of male (*n* = 252) and female (*n* = 248) patients. At the time of preoperative evaluation, 69% of children were attending mainstream school, either with (31%) or without (38%) additional support, and 12% were attending a special educational needs school.

The median age of epilepsy onset was 3.2 years [IQR = (1.2, 6.5)], and the median age at surgery was 11.9 years [IQR = (7.8, 15.0)]. Most children (65%) had a focal MRI abnormality on their preoperative scan, and the remaining children had either a diffuse (23%) or multifocal (8%) MRI abnormality or were diagnosed as MRI negative (4%). Lesionectomy (42%) and lobectomy (35%) were the most commonly performed procedures, followed by hemispherotomy (16%). Most surgeries were performed on the temporal (47%) and frontal (18%) lobes. Low-grade epilepsy-associated tumour (25%), mesial temporal sclerosis (15%) and focal cortical dysplasia type-II (14%) were the most commonly occurring histopathology diagnoses.

At 1-year postoperative follow-up, 66% of patients were seizure free. Of these, 51% were receiving ASMs, 31% were weaning ASMs and 16% were no longer receiving ASMs (2% of the patients who were seizure free were missing data regarding postoperative ASM status).

A comparison of patient characteristics between patients with one versus multiple pre- and/or postoperative assessments, and of surgical patients with and without neuropsychological assessment, is displayed in Supplementary Table 5. Patients with multiple preoperative assessments underwent surgery at an older age [Md_single_ = 11.8 years, IQR_single_ = (7.9, 15.0) versus Md_multi_ = 13.4 years, IQR_multi_ = (10.4, 15.6), *P* = 0.009], had a longer duration of epilepsy [Md_single_ = 6.0 years, IQR_single_ = (3.9, 9.7) versus Md_multi_ = 8.2 years, IQR_multi_ = (5.3, 10.8), *P* = 0.002] and had been trialled on a larger number of ASMs preoperatively (M_single_ = 4.4, SD_single_ = 2.1 versus M_multi_ = 5.5, SD_multi_ = 2.6, *P* < 0.001). Those with multiple preoperative assessments were also more likely to have a non-focal MRI abnormality or to be considered MRI negative [χ^2^(1, 452) = 10.6, *P* = 0.026]. There were no significant differences between patients with a single versus multiple postoperative assessments. There were, however, several noteworthy differences in patient characteristics between the surgical cohorts with versus without neuropsychology assessment.

### Preoperative neuropsychological trajectories

**Figure 1:**
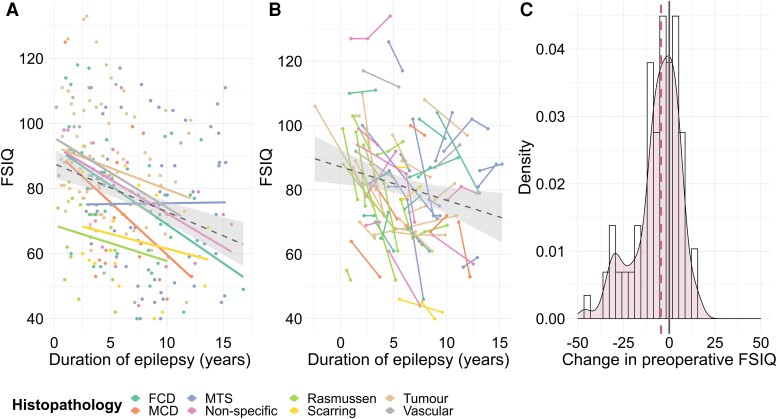
**Preoperative changes in neuropsychological performance.** (**A**) Cross-sectional trajectories in FSIQ across different aetiologies. A single preoperative assessment was included for each patient. If patients had more than one preoperative assessment, the assessment closest to surgery was chosen. Linear modelling was fitted to all data-points (dashed line); shaded area represents the 95% confidence interval. (**B**) Longitudinal trajectories in FSIQ across different aetiologies. Only patients with two or more preoperative assessments were included in the visualization. All preoperative assessments were included for each patient. Linear modelling was fitted to all data-points (dotted line); shaded area represents the 95% confidence interval. One patient, diagnosed with a tumour, had an FSIQ score available prior to epilepsy onset. (**C**) Individual change in preoperative FSIQ. The distribution of change scores is represented using a density plot. The change score for each patient was computed by subtracting the first preoperative score for the patient from their last preoperative score. The median change score is displayed with a dashed purple line. Only patients with two or more preoperative assessments were included in the visualization (patients with unknown aetiology were also included). See Supplementary Fig. 1 for verbal IQ, performance IQ, working memory, processing speed, reading, spelling and numeracy. FCD = focal cortical dysplasia; FSIQ = full-scale IQ; MCD = malformation of cortical development; MTS = mesial temporal sclerosis.

Children showed declines across all eight domains of neuropsychological functioning in the time leading up to surgery (all *P*-values ≤0.001; β_FSIQ_ = −1.9, SE_FSIQ_ = 0.3, *P*_FSIQ_ < 0.001; β_Verbal IQ_ = −1.6, SE_Verbal IQ_ = 0.2, *P*_Verbal IQ_ < 0.001; β_Performance IQ_ = −1.1, SE_Performance IQ_ = 0.2, *P*_Performance IQ_ < 0.001; see Supplementary Table 6 for working memory, processing speed, reading, spelling and numeracy). These declines were observed both cross-sectionally and longitudinally (Fig. 1 for FSIQ; Supplementary Fig. 1 for verbal IQ, performance IQ, working memory, processing speed, reading, spelling and numeracy). These declines were, furthermore, observed in both children with Rasmussen encephalitis and children with non-progressive aetiologies (see Supplementary Fig. 2 for FSIQ, verbal IQ and performance IQ). However, the rates of decline in FSIQ and processing speed were greater in children with Rasmussen encephalitis in comparison to those with non-progressive aetiologies (interaction effect between duration of epilepsy and Rasmussen encephalitis versus non-progressive aetiology: β_FSIQ_ = −2.7, SE_FSIQ_ = 1.1, *P*_FSIQ_ = 0.020; β_Processing Speed_ = −3.4, SE_Processing Speed_ = 1.4, *P*_Processing Speed_ = 0.017; Supplementary Table 6).

In children with more than one preoperative neuropsychology assessment, there was a significant decline in FSIQ, verbal IQ, processing speed and numeracy from their first to their last neuropsychology assessment prior to surgery (all *P*-values ≤0.039; Supplementary Table 7). On average, children lost one to four points per year in the time leading up to their surgery, depending on the domain considered. A significant proportion of children (27%–43%) showed a decline of ≥10 points from their first to last preoperative assessment.

### Neuropsychological performance at presurgical evaluation

By the time children underwent presurgical evaluation, the majority (50%–66%) scored one or more standard deviations below the mean (<85) on the five IQ domains (Supplementary Table 8). Median FSIQ was 75 [IQR = (61, 89); range = 40–133]; one-third of these children (37%) met the threshold for intellectual disability (FSIQ <70). Conversely, one-third of the children (31%–43%) scored within the average range (85–115) on the five IQ domains, and few children (3%–6%) scored above average (>115). A similar pattern was observed for preoperative measures of academic attainment, with half of the children (46%–55%) scoring one or more standard deviations below the mean (<85).

### Change from pre- to postoperative neuropsychological assessment

On a group level, there was no change in performance on any of the measures obtained through neuropsychology assessment, from the last preoperative assessment to the first postoperative assessment (all *P*-values ≥0.128; Supplementary Table 9). On an individual level, most children (54%–71%) showed stable performance across neuropsychology domains from pre- to postoperative assessment. A similar proportion of children showed an improvement (≥10 points; 8%–23%) and decline (≤10 points; 14%–28%) in test scores from pre- to postoperative assessment.

### Postoperative neuropsychological trajectories

**Figure 2:**
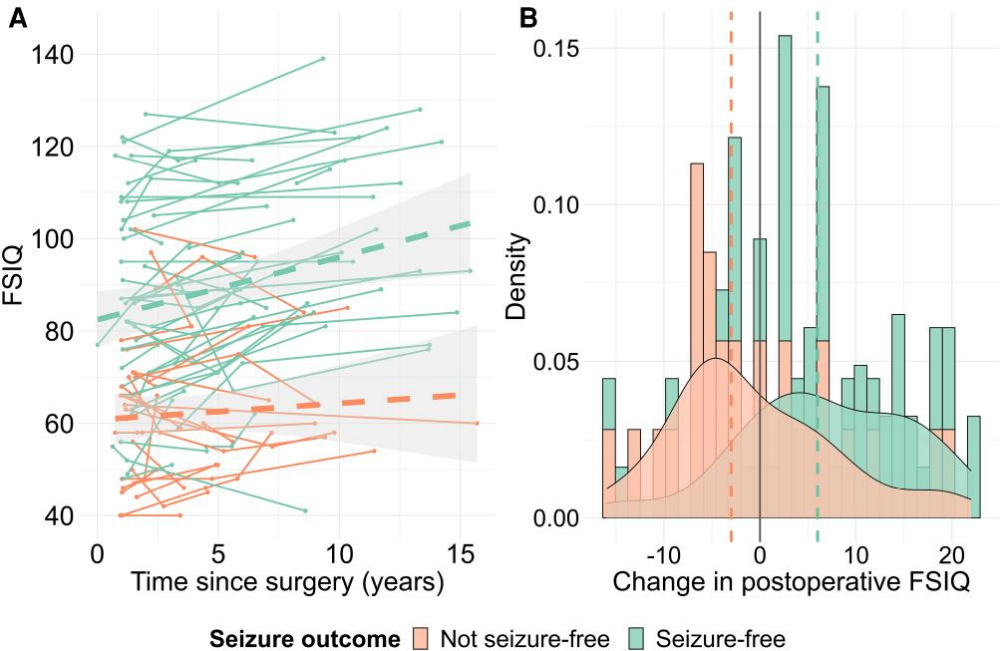
**Postoperative changes in neuropsychological performance.** (**A**) Longitudinal trajectories in FSIQ in seizure-free (green) versus not seizure-free (orange) patients. Only patients with two or more postoperative assessments were included. All postoperative assessments were included for each patient. Linear modelling was fitted to all data-points, according to seizure outcome (dashed green line for patients who were seizure free and dashed orange line for patients who were not seizure free); shaded areas represent the 95% confidence intervals. (**B**) Individual change in postoperative FSIQ in seizure-free (green) versus not seizure-free (orange) patients. The distribution of change scores is represented using a density plot. The change score for each patient was computed by subtracting the first postoperative score for the patient from their last postoperative score. The median change scores are displayed with a green dashed line (in seizure-free patients) and with an orange dashed line (in not seizure-free patients). Only patients with two or more postoperative assessments were included in the visualization. See Supplementary Fig. 3 for verbal IQ, performance IQ, working memory, processing speed, reading, spelling and numeracy. FSIQ = full-scale IQ.

In children with multiple postoperative neuropsychology assessments, there was no overall change in performance from the first to last postoperative assessment of the patients (all *P*-values ≥0.246; Supplementary Table 10). However, children who were rendered seizure free through surgery did improve over the course of the postoperative period on measures of FSIQ, verbal IQ, working memory and reading, whereas children who continued to have seizures either kept declining or plateaued (interaction effect between duration of postoperative follow-up and seizure free versus not seizure free: β_FSIQ_ = 0.9, SE_FSIQ_ = 0.3, *P*_FSIQ_ = 0.004; β_Verbal IQ_ = 1.0, SE_Verbal IQ_ = 0.3, *P*_Verbal IQ_ = 0.003; β_Working memory_ = 1.0, SE_Working memory_ = 0.4, *P*_Working memory_ = 0.010; β_Reading_ = 1.6, SE_Reading_ = 0.5, *P*_Reading_ = 0.002; see Supplementary Table 11 for performance IQ, processing speed, spelling and numeracy; see Fig. 2 for FSIQ; Supplementary Fig. 3 for verbal IQ, performance IQ, working memory, processing speed, reading, spelling and numeracy).

**Figure 3:**
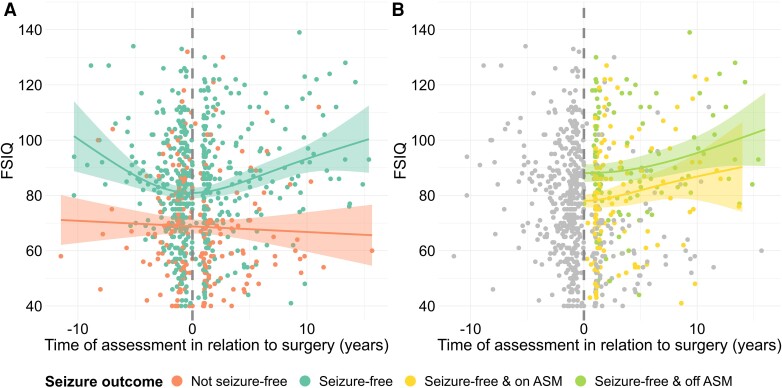
**Pre- to postoperative changes in neuropsychological performance.** (**A**) Pre- and postoperative trajectories in FSIQ in seizure-free (green) versus not seizure-free (orange) patients. All patients were included in the visualization, irrespective of whether they had one or multiple pre- and/or postoperative assessments. If patients had multiple pre- and/or postoperative assessments, all assessments were included for each patient. Generalized additive modelling was fitted to all data-points, according to seizure outcome (solid green line for patients who were seizure free and solid orange line for patients who were not seizure free); shaded areas represent the 95% confidence intervals. (**B**) Pre- and postoperative trajectories in FSIQ in seizure-free patients who were still receiving or weaning ASMs (yellow) versus no longer receiving ASMs (light green). Grey points represent FSIQ in the preoperative period and FSIQ for patients who did not become seizure free postoperatively. All patients were included in the visualization, irrespective of whether they had one or multiple pre- and/or postoperative assessments. If patients had multiple pre- and/or postoperative assessments, all assessments were included for each patient. Generalized additive modelling was fitted to all data-points, according to postoperative ASM status (solid yellow line for patients who were still receiving or weaning ASMs and solid light green line for patients who were no longer receiving ASMs); shaded areas represent the 95% confidence intervals. See Supplementary Fig. 4 for verbal IQ, performance IQ, working memory, processing speed, reading, spelling and numeracy. ASM = antiseizure medication; FSIQ = full-scale IQ.

Pooling all pre- and postoperative neuropsychology data, including multiple assessments from the same child, showed that children who became seizure free declined in functioning preoperatively but improved significantly postoperatively (Fig. 3A for FSIQ; see Supplementary Fig. 4 for verbal IQ, performance IQ, working memory, processing speed, reading, spelling and numeracy). There also seemed to be an added benefit of no longer receiving ASMs, as opposed to still receiving or weaning them (Fig. 3B for FSIQ).

Further analysis of the preoperative trajectory in those with multiple preoperative assessments showed that children who became seizure free postoperatively performed significantly better at both the first and last preoperative assessment in comparison to those who did not become seizure free (Supplementary Fig. 5 for FSIQ). There was, however, no interaction effect between time point of preoperative assessment and seizure outcome [all *P*-values ≥0.414; *F*_FSIQ_(1,158) = 0.4, *P* = 0.511; Supplementary Table 12], suggesting that both groups declined preoperatively. Children who did not become seizure free had a younger age of epilepsy onset, had been trialled on a larger number of different ASMs from the time of epilepsy onset to the time of surgery, were more likely to have a non-focal MRI abnormality and were more likely to have a diagnosis of malformation of cortical development-other, tuberous sclerosis, vascular aetiology or non-specific epilepsy-associated changes (Supplementary Table 13).

### Determinants of neuropsychological performance

Relationships between patient characteristics and neuropsychological functioning are presented in Supplementary Table 14 (including full statistical information).

#### Preoperative neuropsychological trajectories

Decline in neuropsychological functioning over the course of the preoperative trajectory was related to a history of generalized tonic–clonic seizures, a diagnosis of Rasmussen encephalitis and a higher score on initial neuropsychological assessment; however, only a history of generalized tonic–clonic seizures survived correction for multiple comparison.

#### Neuropsychological performance at the time of presurgical evaluation

A lower score on preoperative neuropsychological assessment (closest to surgery) was associated with a younger age of epilepsy onset, a longer duration of epilepsy (even after controlling for age of epilepsy onset), a greater ASM load (both having trialled a larger number of ASMs from time of epilepsy onset to time of surgery and receiving a larger number of ASMs at time of preoperative evaluation), a diagnosis of non-focal MRI abnormality (diffuse or multifocal MRI abnormality or MRI negative) and a diagnosis of Rasmussen encephalitis.

#### Postoperative neuropsychological performance

Postoperative performance (closest to surgery) was highly correlated with preoperative performance (closest to surgery) and therefore related in a similar manner to age of epilepsy onset, ASM load and type of MRI abnormality. Better postoperative performance was additionally related to having undergone a focal resection (lesionectomy or lobectomy procedure as opposed to disconnection, hemispherotomy or corpus callosotomy), a diagnosis of low-grade epilepsy-associated tumour and being seizure free postoperatively.

Children who were seizure free and no longer receiving ASMs at 1-year follow-up had higher working memory (Md_Off ASM_ = 91 versus Md_On ASM/Weaning_ = 83, *P* = 0.026), processing speed (Md_Off ASM_ = 88 versus Md_On ASM/Weaning_ = 80, *P* = 0.012) and numeracy (Md_Off ASM_ = 94 versus Md_On ASM/Weaning_ = 81, *P* = 0.028) at first postoperative assessment in comparison to seizure-free patients who were still receiving or weaning ASMs (Supplementary Table 15).

#### Change from pre- to postoperative neuropsychological assessment

An improvement in neuropsychological functioning from pre- to postoperative assessment was related to lower preoperative neuropsychological performance, older age at surgery and longer duration of epilepsy at the time of surgery. Seizure freedom was also related to an improvement in neuropsychological functioning from pre- to postoperative assessment, but only at last postoperative follow-up and not at first postoperative follow-up.

#### Postoperative neuropsychological trajectories

As observed in the abovementioned individual postoperative trajectories, improvement in neuropsychological performance over the postoperative period was associated with being seizure free postoperatively. An improvement in performance IQ was also related to having undergone a focal resection (lesionectomy or lobectomy procedure as opposed to disconnection, hemispherotomy or corpus callosotomy).

Patients who were seizure free and no longer receiving ASMs at final follow-up showed greater improvement in verbal IQ, working memory, reading and spelling over the course of the postoperative period compared with seizure-free patients who were still receiving or weaning ASMs (all *P*-values ≤0.034; Supplementary Table 16).

## Discussion

This is the first study to characterize long-term neuropsychological trajectories in children with drug-resistant epilepsy, both before and after surgical intervention. Past studies that have investigated neuropsychological functioning in paediatric epilepsy surgery patients have typically included a single pre- and postoperative assessment, obtained directly before and 1 year after surgery, respectively. In this study, we included multiple assessments obtained throughout the preoperative period, in addition to multiple postoperative assessments. We found that children showed declines across all domains of neuropsychological functioning in the time leading up to surgery. However, we also observed that children who became seizure free through surgery displayed significant recoveries in neuropsychological functioning, both at the time of initial follow-up and throughout the course of the postoperative period.

### Children with epilepsy undergoing surgery show preoperative declines in neuropsychological functioning

We found that children showed significant declines in functioning across multiple domains of neuropsychological functioning in the time leading up to surgery. This was true for children with Rasmussen encephalitis and for children with non-progressive aetiologies. However, children with Rasmussen encephalitis showed greater declines in FSIQ and processing speed in comparison to those with non-progressive aetiologies. Nevertheless, we found it surprising that the decline observed in children with non-progressive aetiologies was largely comparable to that of children with Rasmussen encephalitis, where the cognitive decline in the disease has been associated with a decline in grey matter volume.^64^ This might suggest that the mechanism(s) of cognitive decline is shared across epilepsy aetiologies and could involve ongoing seizures, interictal discharges and the use of ASMs.^3^

We found that a number of additional clinical characteristics were related to a greater preoperative decline, including a history of generalized tonic–clonic seizures and a higher score on initial neuropsychological assessment. However, only a history of generalized tonic–clonic seizures survived correction for multiple comparison. Frequency of generalized tonic–clonic seizures has previously been found to be the strongest predictor of cognitive decline in adults with epilepsy.^65^

By the time children underwent preoperative evaluation, the majority (46%–66%) showed impairments across neuropsychological domains. A significant proportion of children (43%) were attending a special education needs school or receiving educational support in mainstream school. This is fully in line with past studies that have demonstrated that neuropsychological impairments are common in children undergoing presurgical evaluation for epilepsy surgery.^52^

Lower neuropsychological performance at the time of presurgical evaluation was associated with a range of clinical characteristics, including younger age of epilepsy onset. This could indicate that, if the epilepsy emerges early in the still-maturing brain, it could impact on cognitive development disproportionally.^66^ Independently of this effect of age of epilepsy onset, we found that a longer duration of epilepsy further impacted negatively upon neuropsychological abilities, calling for early intervention.

### Seizure freedom is associated with postoperative improvement in neuropsychological functioning

Across all patients, there was no significant change in neuropsychological performance from pre- to postoperative assessment, which mirrors findings from past studies.^8,9,11,12,14,19,20,22,23,25,27-43^

Higher postoperative neuropsychological performance was strongly correlated with higher preoperative performance and therefore also older age of epilepsy onset. This might suggest that the establishment of a form of normal baseline cognition prior to surgery could act as a foundation for postoperative skills.^59^ Patients with lower neuropsychological functioning at the time of preoperative assessment (most probably attributable to earlier age of epilepsy onset and longer duration of epilepsy) were more likely to improve from pre- to postoperative neuropsychological assessment. This ‘catch-up’ effect in functioning could be driven by postoperative structural^17,26^ and functional^67^ brain plasticity. At the same time, one might argue that patients with the highest neuropsychological functioning at the time of preoperative assessment have ‘the most to lose’, as previously proposed.^17,67^

**Figure 4:**
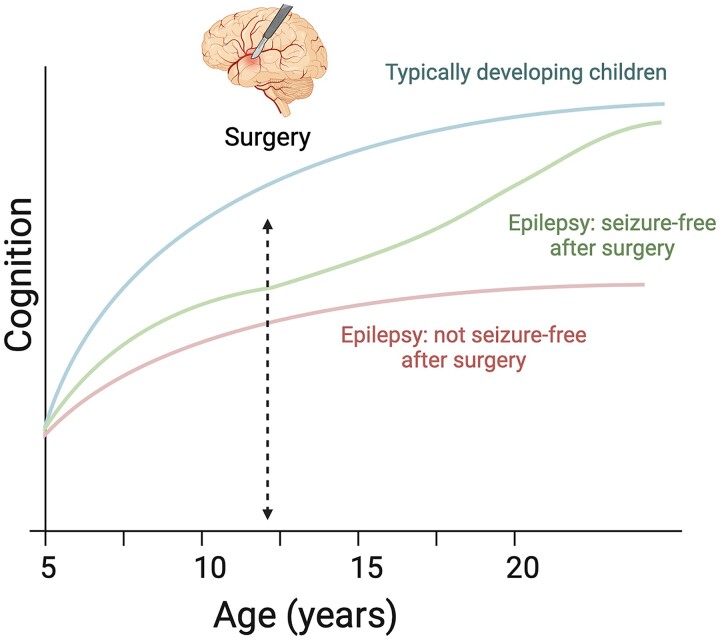
**Overview of study findings.** Based on our findings, we propose the following schematic model to capture the impact of epilepsy surgery on neuropsychological trajectories in children with drug-resistant focal epilepsy. Prior to epilepsy surgery, children with epilepsy fall increasingly behind their typically developing peers, resulting in them displaying a decline in IQ (standard) scores. Children who are rendered seizure free after surgery display a ‘catching-up’ effect, which allows them to attain a cognitive level that is comparable to that of their typically developing peers without epilepsy. Children who are not seizure free after surgery do not display this same ‘catching-up’ effect and have lower IQ scores already prior to surgery. The blue line (of typically developing children) shows the mean of a normative sample and corresponds to an IQ score of 100 at each data-point.

Importantly, we found that postoperative seizure freedom was associated with better postoperative neuropsychological functioning. Observing the postoperative trajectories, seizure freedom also seemed to provide the opportunity for neuropsychological abilities to recover further over time (Fig. 4). Although a significant number of past studies have also found that seizure freedom is associated with better postoperative neuropsychological performance or an improvement in performance from pre- to postoperative assessment,^18,20-22,37,39,40,50^ a similar number of studies have failed to find an association between seizure outcome and postoperative neuropsychological performance.^9,10,14,17,24,26,33,35,48,49^ These contradictory findings might be explained, in part, by the varying length of follow-up adopted by the individual studies. Most studies conducted to date have reported neuropsychological outcomes in the first few years after surgery. It has, however, also been suggested^22,26^ that this form of short-term follow-up is not sufficient to allow for the manifestation of cognitive recovery. Indeed, we found that seizure freedom was significantly associated with an improvement in neuropsychological functioning from pre- to postoperative assessment only at final follow-up, and not at first follow-up. Furthermore, visual inspection of the postsurgical trajectories in the present study revealed that further improvement in neuropsychological functioning in seizure-free patients did not appear to emerge until >5 years after surgery and became more pronounced with time. This is in keeping with the notion of a protracted normalization of brain networks postoperatively.^68^

Finally, we found that no longer receiving ASMs (as opposed to still receiving or being in the process of being weaned off ASMs) seemed to provide an additional benefit to neuropsychological functioning above simply being seizure free, both at the time of 1-year postoperative follow-up and over the course of the postoperative period. This mirrors the findings of single-centre studies^21,52^ and of a European multicentre study.^49^

Taken together, this suggests that, by providing seizure freedom and the opportunity to discontinue ASM treatment, epilepsy surgery has the potential not only to halt but to reverse the downward trajectory in neuropsychological functioning that children with drug-resistant epilepsy experience. Therefore, to prevent as much of this decline as possible, children should be referred and evaluated for epilepsy surgery as early as possible.

### A common factor underlying neuropsychological performance and the likelihood of seizure freedom?

The difference in neuropsychological performance between patients who became seizure free through surgery and those who did not was already apparent at the time of preoperative assessment. Specifically, patients who did not become seizure free showed lower neuropsychological functioning on their first and last preoperative assessment in comparison to patients who became seizure free (although both groups declined in functioning preoperatively). This might suggest that there is an underlying factor driving both cognitive development and the likelihood of patients becoming seizure free through surgery. The exact nature of this underlying factor remains uncertain; however, it might involve disruptions to regional and remote brain networks.^69-71^ It might therefore be advantageous to incorporate specific preoperative neuropsychological scores, or trajectories, in the prediction of postoperative seizure freedom. The presence of cognitive impairment has been found to be an important predictor of seizure outcome.^72,73^ We have also previously used educational status, which is in itself reflective of neuropsychological functioning, as a feature in a predictive model of seizure outcome^74^; however, there might be added value in including more fine-grained neuropsychological information in the form of test scores to a predictive model of seizure outcome.

It is worth highlighting that the spread in preoperative IQ scores observed amongst patients who were rendered seizure free through surgery was wide. In other words, a low preoperative IQ did not preclude the patient from achieving seizure freedom through surgery. Low preoperative IQ should therefore not be taken as a contraindication to evaluation for epilepsy surgery.^73^

### Limitations of the present study

The primary limitations of this study are the retrospective study design and the patient inclusion criteria.

#### The methodological constraints of clinical data

The epilepsy characteristics that we included in the present study were extracted from medical records and used to describe either historical information (e.g. whether the patient had a history of infantile spasms) or information obtained at the time of presurgical evaluation (e.g. the number of ASMs prescribed at the time of presurgical evaluation). Only duration of epilepsy was additionally obtained during the time of neuropsychological assessment. Ideally, these epilepsy characteristics, in particular those relating to seizure types and frequency and ASM load, should be collected prospectively and concurrently in relationship to the neuropsychology assessment(s) of the patients. The lack of temporal relationship between these epilepsy characteristics and preoperative decline in neuropsychological functioning might explain why many of the associations that we identified did not survive correction for multiple comparisons.

Furthermore, the data included in this study were originally collected for clinical purposes and not in accordance with a standardized research protocol. Although neuropsychological assessments are standardized in their structure and administration, some degree of tailoring might be needed to take into account the needs and priorities of individual patients and potential comorbidities. As a result, not every cognitive subtest and/or academic measure was administrated to each patient. We therefore had a varying number of data-points available across the different neuropsychological domains.

There are currently efforts to collect neuropsychological data prospectively for epilepsy surgery patients across multiple sites in the USA.^75^ These will represent an invaluable resource for clinical decision-making in the future. It might also be important to include measures of preoperative functional brain mapping, because patients in whom eloquent brain areas were resected have been found to have worse neuropsychological outcomes compared with those in whom eloquent areas were preserved.^76^

#### Sources of selection bias

Another limitation of the present study is the way that the cohort was selected. We aimed to collate a surgical cohort that was diverse in terms of the types of surgery that patients had undergone, in addition to the underlying aetiologies of their epilepsies. Nonetheless, we included only patients who had undergone neuropsychological assessment using age-appropriate IQ tests. We thus excluded surgical patients who were unable to access these tests or who lacked these data for other reasons, which might, in turn, have given rise to selection bias. Indeed, when we compared the clinical characteristics of the patients included in this study with the overall cohort of patients who had undergone epilepsy surgery during the study time period, we found several significant differences in the clinical characteristics between the two cohorts. These differences suggested that the surgical patients who had not undergone neuropsychological assessment were younger, suffered from a greater degree of epilepsy severity and were less able developmentally. As a result, we are only able to comment on the developmental trajectories of older, more able children undergoing epilepsy surgery. For this reason, it is also likely that the study underestimated the frequency and severity of cognitive impairment in paediatric epilepsy surgery patients. Past studies^24,59^ have investigated the impact of epilepsy surgery on developmental outcomes in younger and/or less able patients using DQ scores; however, there are considerable methodological difficulties associated with equating or combining DQ and IQ scores.^57,58^ It has also been shown that there are different determinants of both pre- and postoperative IQ and DQ in patients undergoing epilepsy surgery.^59^ For example, Cloppenborg *et al*.^59^ found that older age at surgery was associated with better postoperative IQ, whereas the opposite effect was found for postoperative DQ in the same study. It is therefore arguably better to treat these two cohorts as separate groups.

In discussing selection bias, it is also important to remember that the study cohort was derived from a tertiary epilepsy surgery centre. These children are more likely to have a higher degree of epilepsy severity (and drug resistance) in comparison to children with ‘uncomplicated’ epilepsy. The findings of the study relating to the neuropsychological trajectories of children with epilepsy, in particular the decline in functioning over the course of disease duration, might therefore not be generalizable to other epilepsy patient populations. Indeed, a large-scale community-based study showed that children with drug-resistant epilepsy have significantly lower IQ scores in comparison to those who are not drug resistant.^77^

Finally, one might assume that children who have undergone neuropsychological assessment on more than one occasion, either before or after surgery, are experiencing a more severe form of epilepsy and/or degree of cognitive impairment. However, when we compared the clinical characteristics of those who underwent testing once versus multiple times preoperatively, we did not find noteworthy differences in clinical characteristics between the two groups of patients, with the exception that those who had undergone multiple assessments had a longer duration of epilepsy and, perhaps in extension, had been trialled on a larger number of ASMs since their epilepsy onset. Reassuringly, there were no significant differences in clinical characteristics between patients who had undergone a single versus multiple neuropsychological assessments postoperatively, including no differences in seizure outcome or neuropsychological functioning at initial postoperative follow-up. It is therefore reasonable to assume that the findings relating to patients with multiple assessments might generalize well to those who had undergone a single assessment.

## Conclusions

Children with drug-resistant epilepsy show significant declines in neuropsychological functioning in the time leading up to surgery. Epilepsy surgery has the potential to provide these children with seizure freedom and, in extension, reverse their downward neuropsychological trajectory. However, to minimize these declines, or potentially prevent them from occurring in the first place, children with focal epilepsy should be referred and evaluated for epilepsy surgery as early as possible.

## Supplementary Material

awae121_Supplementary_Data

## Data Availability

The study’s data dictionary and analytic code will be made publicly available on GitHub (https://github.com/MariaEriksson/Neuropsychological-trajectories-paper). The full data are not publicly available owing to their sensitive nature. Deidentified data will be made available from the corresponding author upon reasonable request.

## References

[1] Nickels KC , Zaccariello MJ, Hamiwka LD, Wirrell EC. Cognitive and neurodevelopmental comorbidities in paediatric epilepsy. Nat Rev Neurol. 2016;12:465–476. 27448186 10.1038/nrneurol.2016.98

[2] Camfield CS , Camfield PR. Long-term social outcomes for children with epilepsy. Epilepsia. 2007;48:3–5. 10.1111/j.1528-1167.2007.01390.x 18047590

[3] Braun KPJ . Preventing cognitive impairment in children with epilepsy. Curr Opin Neurol. 2017;30:140–147. 28141740 10.1097/WCO.0000000000000424

[4] Hermann B , Meador KJ, Gaillard WD, Cramer JA. Cognition across the lifespan: Antiepileptic drugs, epilepsy, or both? Epilepsy Behav. 2010;17:1–5. 19931492 10.1016/j.yebeh.2009.10.019

[5] Cross JH , Reilly C, Gutierrez Delicado E, Smith ML, Malmgren K. Epilepsy surgery for children and adolescents: Evidence-based but underused. Lancet Child Adolesc Health. 2022;6:484–494. 35568054 10.1016/S2352-4642(22)00098-0

[6] Eriksson MH , Whitaker KJ, Booth J, et al Pediatric epilepsy surgery from 2000 to 2018: Changes in referral and surgical volumes, patient characteristics, genetic testing, and postsurgical outcomes. Epilepsia. 2023;64:2260–2273. 37264783 10.1111/epi.17670 PMC7615891

[7] Widjaja E , Jain P, Demoe L, Guttmann A, Tomlinson G, Sander B. Seizure outcome of pediatric epilepsy surgery: Systematic review and meta-analyses. Neurology. 2020;94:311–321. 31996452 10.1212/WNL.0000000000008966

[8] Beaton AE , Durnford A, Heffer-Rahn PE, Kirkham F, Griffin A, Gray WLS. Transsylvian selective amygdalohippocampectomy in children with hippocampal sclerosis: Seizure, intellectual and memory outcome. Seizure. 2012;21:699–705. 22898674 10.1016/j.seizure.2012.07.012

[9] Clusmann H , Kral T, Gleissner U, et al Analysis of different types of resection for pediatric patients with temporal lobe epilepsy. Neurosurgery. 2004;54:847–860. 15046650 10.1227/01.neu.0000114141.37640.37

[10] Freitag H , Tuxhorn I. Cognitive function in preschool children after epilepsy surgery: Rationale for early intervention. Epilepsia. 2005;46:561–567. 15816951 10.1111/j.0013-9580.2005.03504.x

[11] García-Fernández M , Fournier-Del Castillo C, Ugalde-Canitrot A, et al Epilepsy surgery in children with developmental tumours. Seizure. 2011;20:616–627. 21741275 10.1016/j.seizure.2011.06.003

[12] Jambaqué I , Dellatolas G, Fohlen M, et al Memory functions following surgery for temporal lobe epilepsy in children. Neuropsychologia. 2007;45:2850–2862. 17612579 10.1016/j.neuropsychologia.2007.05.008

[13] Liu S , An N, Yang H, et al Pediatric intractable epilepsy syndromes: Reason for early surgical intervention. Brain Dev. 2007;29:69–78. 16930902 10.1016/j.braindev.2006.06.009

[14] Mabbott DJ , Smith ML. Memory in children with temporal or extra-temporal excisions. Neuropsychologia. 2003;41:995–1007. 12667535 10.1016/s0028-3932(02)00318-4

[15] Meekes J , Braams O, Braun KPJ, Jennekens-Schinkel A, Van Nieuwenhuizen O. Verbal memory after epilepsy surgery in childhood. Epilepsy Res. 2013;107(1–2):146–155. 24042124 10.1016/j.eplepsyres.2013.08.017

[16] Miserocchi A , Cascardo B, Piroddi C, et al Surgery for temporal lobe epilepsy in children: Relevance of presurgical evaluation and analysis of outcome. J Neurosurg Pediatr. 2013;11:256–267. 23311387 10.3171/2012.12.PEDS12334

[17] Skirrow C , Cross JH, Cormack F, Harkness W, Vargha-Khadem F, Baldeweg T. Long-term intellectual outcome after temporal lobe surgery in childhood. Neurology. 2011;76:1330–1337. 21482948 10.1212/WNL.0b013e31821527f0 PMC3090063

[18] Skirrow C , Cross JH, Harrison S, et al Temporal lobe surgery in childhood and neuroanatomical predictors of long-term declarative memory outcome. Brain. 2015;138:80–93. 25392199 10.1093/brain/awu313 PMC4285190

[19] Van Oijen M , De Waal H, Van Rijen PC, Jennekens-Schinkel A, Van Huffelen AC, Van Nieuwenhuizen O. Resective epilepsy surgery in childhood: The Dutch experience 1992–2002. Eur J Paediatr Neurol. 2006;10:114–123. 16769233 10.1016/j.ejpn.2006.04.003

[20] Lendt M , Helmstaedter C, Elger CE. Pre- and postoperative neuropsychological profiles in children and adolescents with temporal lobe epilepsy. Epilepsia. 1999;40:1543–1550. 10565581 10.1111/j.1528-1157.1999.tb02038.x

[21] Helmstaedter C , Elger CE, Witt JA. The effect of quantitative and qualitative antiepileptic drug changes on cognitive recovery after epilepsy surgery. Seizure. 2016;36:63–69. 26954934 10.1016/j.seizure.2016.02.001

[22] Puka K , Tavares TP, Smith ML. Development of intelligence 4 to 11 years after paediatric epilepsy surgery. J Neuropsychol. 2017;11:161–173. 26184054 10.1111/jnp.12081

[23] Smith ML , Olds J, Snyder T, Elliott I, Lach L, Whiting S. A follow-up study of cognitive function in young adults who had resective epilepsy surgery in childhood. Epilepsy Behav. 2014;32:79–83. 24508594 10.1016/j.yebeh.2014.01.006

[24] Loddenkemper T , Holland KD, Stanford LD, Kotagal P, Bingaman W, Wyllie E. Developmental outcome after epilepsy surgery in infancy. Pediatrics. 2007;119:930–935. 17473093 10.1542/peds.2006-2530

[25] Hermann BP , Seidenberg M, Haltiner A, Wyler AR. Relationship of age at onset, chronologic age, and adequacy of preoperative performance to verbal memory change after anterior temporal lobectomy. Epilepsia. 1995;36:137–145. 7821270 10.1111/j.1528-1157.1995.tb00972.x

[26] Skirrow C , Cross JH, Owens R, et al Determinants of IQ outcome after focal epilepsy surgery in childhood: A longitudinal case-control neuroimaging study. Epilepsia. 2019;60:872–884. 30968956 10.1111/epi.14707

[27] De Koning T , Versnel H, Jennekens-Schinkel A, et al Language development before and after temporal surgery in children with intractable epilepsy. Epilepsia. 2009;50:2408–2419. 19674043 10.1111/j.1528-1167.2009.02264.x

[28] Gleissner U , Sassen R, Lendt M, Clusmann H, Elger CE, Helmstaedter C. Pre- and postoperative verbal memory in pediatric patients with temporal lobe epilepsy. Epilepsy Res. 2002;51:287–296. 12399079 10.1016/s0920-1211(02)00158-4

[29] Korkman M , Granström ML, Kantola-Sorsa E, et al Two-year follow-up of intelligence after pediatric epilepsy surgery. Pediatr Neurol. 2005;33:173–178. 16139731 10.1016/j.pediatrneurol.2005.04.006

[30] Lah S , Smith ML. Verbal memory and literacy outcomes one year after pediatric temporal lobectomy: A retrospective cohort study. Epilepsy Behav. 2015;44:225–233. 25771353 10.1016/j.yebeh.2014.12.040

[31] Lee YJ , Kang HC, Bae SJ, et al Comparison of temporal lobectomies of children and adults with intractable temporal lobe epilepsy. Childs Nerv Syst. 2010;26:177–183. 19902220 10.1007/s00381-009-1015-3

[32] Lee YJ , Kang HC, Kim HD, et al Neurocognitive function in children after anterior temporal lobectomy with amygdalohippocampectomy. Pediatr Neurol. 2015;52:88–93. 25439484 10.1016/j.pediatrneurol.2014.09.006

[33] Smith ML , Elliott IM, Lach L. Memory outcome after pediatric epilepsy surgery: Objective and subjective perspectives. Child Neuropsychol. 2006;12:151–164. 16837391 10.1080/09297040591001076

[34] Sinclair DB , Aronyk K, Snyder T, et al Pediatric temporal lobectomy for epilepsy. Pediatr Neurosurg. 2003;38:195–205. 12646739 10.1159/000069099

[35] Smith ML , Elliott IM, Lach L. Cognitive, psychosocial, and family function one year after pediatric epilepsy surgery. Epilepsia. 2004;45:650–660. 15144430 10.1111/j.0013-9580.2004.21903.x

[36] Vadera S , Kshettry VR, Klaas P, Bingaman W. Seizure-free and neuropsychological outcomes after temporal lobectomy with amygdalohippocampectomy in pediatric patients with hippocampal sclerosis. J Neurosurg Pediatr. 2012;10:103–107. 22725696 10.3171/2012.4.PEDS1233

[37] Viggedal G , Olsson I, Carlsson G, Rydenhag B, Uvebrant P. Intelligence two years after epilepsy surgery in children. Epilepsy Behav. 2013;29:565–570. 24201119 10.1016/j.yebeh.2013.10.012

[38] Ramantani G , Kadish NE, Mayer H, et al Frontal lobe epilepsy surgery in childhood and adolescence: Predictors of long-term seizure freedom, overall cognitive and adaptive functioning. Neurosurgery. 2018;83:93–103. 29106684 10.1093/neuros/nyx340

[39] Gleissner U , Sassen R, Schramm J, Elger CE, Helmstaedter C. Greater functional recovery after temporal lobe epilepsy surgery in children. Brain. 2005;128:2822–2829. 16014650 10.1093/brain/awh597

[40] Ramantani G , Kadish NE, Brandt A, et al Seizure control and developmental trajectories after hemispherotomy for refractory epilepsy in childhood and adolescence. Epilepsia. 2013;54:1046–1055. 23506137 10.1111/epi.12140

[41] Kuehn SM , Keene DL, Richards PM, Ventureyra EC. Are there changes in intelligence and memory functioning following surgery for the treatment of refractory epilepsy in childhood? Childs Nerv Syst. 2002;18:306–310. 12172937 10.1007/s00381-002-0599-7

[42] Lah SS , Joy P, Bakker K, Miller L. Verbal memory and IQ in children who undergo focal resection for intractable epilepsy: A clinical review. Brain Impair. 2002;3:114–121.

[43] Shurtleff HA , Barry D, Firman T, et al Impact of epilepsy surgery on development of preschool children: Identification of a cohort likely to benefit from early intervention. J Neurosurg Pediatr. 2015;16:383–392. 26140458 10.3171/2015.3.PEDS14359

[44] Vasilica AM , Winsor A, Chari A, Scott R, Baldeweg T, Tisdall M. The influence of disease course and surgery on quality of life in children with focal cortical dysplasia and long-term epilepsy-associated tumours: A systematic review and meta-analysis. Epilepsy Res. 2023;192:107132. 37023554 10.1016/j.eplepsyres.2023.107132

[45] Van Schooneveld MMJ , Braun KPJ. Cognitive outcome after epilepsy surgery in children. Brain and Development. 2013;35:721–729. 23434294 10.1016/j.braindev.2013.01.011

[46] Moosa ANV , Wyllie E. Cognitive outcome after epilepsy surgery in children. Semin Pediatr Neurol. 2017;24:331–339. 29249513 10.1016/j.spen.2017.10.010

[47] Ramantani G , Reuner G. Cognitive development in pediatric epilepsy surgery. Neuropediatrics. 2018;49:093–103. 10.1055/s-0037-1609034 29207404

[48] Puka K , Rubinger L, Chan C, Smith ML, Widjaja E. Predictors of intellectual functioning after epilepsy surgery in childhood: The role of socioeconomic status. Epilepsy Behav. 2016;62:35–39. 27448241 10.1016/j.yebeh.2016.06.023

[49] Boshuisen K , Van Schooneveld MMJ, Uiterwaal CSPM, et al Intelligence quotient improves after antiepileptic drug withdrawal following pediatric epilepsy surgery. Ann Neurol. 2015;78:104–114. 25899932 10.1002/ana.24427

[50] Laguitton V , Desnous B, Lépine A, et al Intellectual outcome from 1 to 5 years after epilepsy surgery in 81 children and adolescents: A longitudinal study. Seizure. 2021;91:384–392. 34298457 10.1016/j.seizure.2021.07.010

[51] Arzimanoglou A , Cross JH, Gaillard WD, et al Pediatric epilepsy surgery. John Libbey Eurotext; 2016. https://books.google.co.uk/books? id=ZtYyDwAAQBAJ.

[52] Helmstaedter C , Beeres K, Elger CE, Kuczaty S, Schramm J, Hoppe C. Cognitive outcome of pediatric epilepsy surgery across ages and different types of surgeries: A monocentric 1-year follow-up study in 306 patients of school age. Seizure. 2020;77:86–92. 31375336 10.1016/j.seizure.2019.07.021

[53] Johnston MV . Clinical disorders of brain plasticity. Brain Dev. 2004;26:73–80. 15036425 10.1016/S0387-7604(03)00102-5

[54] Cross JH , Jayakar P, Nordli D, et al Proposed criteria for referral and evaluation of children for epilepsy surgery: Recommendations of the subcommission for pediatric epilepsy surgery. Epilepsia. 2006;47:952–959. 16822241 10.1111/j.1528-1167.2006.00569.x

[55] Kaufman AS , Flanagan DP, Alfonso VC, Mascolo JT. Test review: Wechsler intelligence scale for children, fourth edition (WISC-IV). J Psychoeduc Assess. 2006;24:278–295.

[56] Busch RM , Lineweaver TT, Ferguson L, Haut JS. Reliable change indices and standardized regression-based change score norms for evaluating neuropsychological change in children with epilepsy. Epilepsy Behav. 2015;47:45–54. 26043163 10.1016/j.yebeh.2015.04.052 PMC4475419

[57] Månsson J , Stjernqvist K, Serenius F, Ådén U, Källén K. Agreement between Bayley-III measurements and WISC-IV measurements in typically developing children. J Psychoeduc Assess. 2019;37:603–616.

[58] Rasheed MA , Kvestad I, Shaheen F, Memon U, Strand TA. The predictive validity of Bayley scales of infant and toddler development-III at 2 years for later general abilities: Findings from a rural, disadvantaged cohort in Pakistan. PLoS Glob Public Health. 2023;3:e0001485. 36962863 10.1371/journal.pgph.0001485 PMC10021670

[59] Cloppenborg T , van Schooneveld M, Hagemann A, et al Development and validation of prediction models for developmental and intellectual outcome following pediatric epilepsy surgery. Neurology. 2022;98:e225–e235. 34795046 10.1212/WNL.0000000000013065

[60] American Psychiatric Association . Diagnostic and statistical manual of mental disorders: DSM-5. 5th edn. American Psychiatric Association; 2013.

[61] Razali NM , Wah YB. Power comparisons of Shapiro-Wilk, Kolmogorov-Smirnov, Lilliefors and Anderson-Darling tests. J Stat Model Anal. 2011;2:21–33.

[62] Varadkar S , Bien CG, Kruse CA, et al Rasmussen’s encephalitis: Clinical features, pathobiology, and treatment advances. Lancet Neurol. 2014;13:195–205. 24457189 10.1016/S1474-4422(13)70260-6 PMC4005780

[63] Aickin M , Gensler H. Adjusting for multiple testing when reporting research results: The Bonferroni vs Holm methods. Am J Public Health. 1996;86:726–728. 8629727 10.2105/ajph.86.5.726 PMC1380484

[64] Rudebeck SR , Shavel-Jessop S, Varadkar S, et al Pre- and postsurgical cognitive trajectories and quantitative MRI changes in Rasmussen syndrome. Epilepsia. 2018;59:1210–1219. 29750339 10.1111/epi.14192

[65] Thompson PJ , Duncan JS. Cognitive decline in severe intractable epilepsy. Epilepsia. 2005;46:1780–1787. 16302858 10.1111/j.1528-1167.2005.00279.x

[66] Sullivan AW , Johnson MK, Boes AD, Tranel D. Implications of age at lesion onset for neuropsychological outcomes: A systematic review focusing on focal brain lesions. Cortex. 2023;163:92–122. 37086580 10.1016/j.cortex.2023.03.002 PMC10192019

[67] Arski ON , Martire DJ, Young JM, et al Connectomic profiles and cognitive trajectories after epilepsy surgery in children. Neurology. 2022;98:e2233–e2244. 35410904 10.1212/WNL.0000000000200273 PMC9162161

[68] Kundu B , Charlebois CM, Anderson DN, Peters A, Rolston JD. Chronic intracranial recordings after resection for epilepsy reveal a “running down” of epileptiform activity. Epilepsia. 2023;64:e135–e142. 37163225 10.1111/epi.17645 PMC10524582

[69] Ailion AS , You X, Mbwana JS, et al Functional connectivity as a potential mechanism for language plasticity. Neurology. 2022;98:e249–e259. 34795045 10.1212/WNL.0000000000013071 PMC8792810

[70] Liu W , Yue Q, Gong Q, Zhou D, Wu X. Regional and remote connectivity patterns in focal extratemporal lobe epilepsy. Ann Transl Med. 2021;9:1128–1128. 34430569 10.21037/atm-21-1374 PMC8350670

[71] Holmes GL . Cognitive impairment in epilepsy: The role of network abnormalities. Epileptic Disord. 2015;17:101–116. 25905906 10.1684/epd.2015.0739 PMC5410366

[72] Castro-Villablanca F , Moeller F, Pujar S, et al Seizure outcome determinants in children after surgery for single unilateral lesions on magnetic resonance imaging: Role of preoperative ictal and interictal electroencephalography. Epilepsia. 2022;63:3168–3179. 36177545 10.1111/epi.17425

[73] Reinholdson J , Olsson I, Edelvik Tranberg A, Malmgren K. Low IQ predicts worse long-term seizure outcome after resective epilepsy surgery—A propensity score matched analysis. Epilepsy Res. 2023;191:107110. 36821876 10.1016/j.eplepsyres.2023.107110

[74] Eriksson MH , Ripart M, Piper RJ, et al Predicting seizure outcome after epilepsy surgery: Do we need more complex models, larger samples, or better data? Epilepsia. 2023;64:2014–2026. 37129087 10.1111/epi.17637 PMC10952307

[75] Berl MM , Koop JI, Ailion A, et al Leveraging expertise and optimizing clinical research: Initial success of a pediatric epilepsy surgery collaborative. Epilepsia. 2023;64:1554–1567. 36897767 10.1111/epi.17579

[76] Sonoda M , Rothermel R, Carlson A, et al Naming-related spectral responses predict neuropsychological outcome after epilepsy surgery. Brain. 2022;145:517–530. 35313351 10.1093/brain/awab318 PMC9014727

[77] Berg AT , Zelko FA, Levy SR, Testa FM. Age at onset of epilepsy, pharmacoresistance, and cognitive outcomes: A prospective cohort study. Neurology. 2012;79:1384–1391. 22972641 10.1212/WNL.0b013e31826c1b55 PMC3448745

