# Supplementary material for: Long-term neuropsychological trajectories in children with epilepsy: does surgery halt decline?
Source: Brain. 2024 Apr 20;147(8):2791–802. doi: 10.1093/brain/awae121 (PMC11292899; doi:10.1093/brain/awae121)
Supplement: awae121_Supplementary_Data [file awae121_supplementary_data.pdf]

Supplementary material associated with the manuscript, "**Long-term neuropsychological trajectories in children with epilepsy: Does surgery halt decline?**"

Supplementary Table 1. Neuropsychological tests.

**Test**	**Test version**	**Neuropsychological domain**	**Test subscale**
**WISC**	**V**	FSIQ	Full-Scale IQ
		Verbal IQ	Verbal Comprehension Index
		Performance IQ	Fluid Reasoning Index
		Working Memory	Working Memory Index
		Processing Speed	Processing Speed Index
**WISC**	**IV**	FSIQ	Full-Scale IQ
		Verbal IQ	Verbal Comprehension Index
		Performance IQ	Perceptual Reasoning Index
		Working Memory	Working Memory Index
		Processing Speed	Processing Speed Index
**WISC**	**III**	FSIQ	Full-Scale IQ
		Verbal IQ	Verbal Comprehension Index [a]
		Performance IQ	Perceptual Organisation Index [b]
		Working Memory	Working Memory Index [c]
		Processing Speed	Processing Speed Index
**WPPSI**	**R**	FSIQ	Full-Scale IQ
		Verbal IQ	Verbal IQ
		Performance IQ	Performance IQ
		Working Memory	-
		Processing Speed	-
**WPPSI**	**IV**	FSIQ	Full-Scale IQ
		Verbal IQ	Verbal Comprehension Index
		Performance IQ	Fluid Reasoning Index
		Working Memory	Working Memory Index
		Processing Speed	Processing Speed Index
**WPPSI**	**III**	FSIQ	Full-Scale IQ
		Verbal IQ	Verbal IQ
		Performance IQ	Performance IQ
		Working Memory	-
		Processing Speed	Processing Speed Quotient
**WAIS**	**IV**	FSIQ	Full-Scale IQ
		Verbal IQ	Verbal Comprehension Index
		Performance IQ	Perceptual Reasoning Index
		Working Memory	Working Memory Index
		Processing Speed	Processing Speed Index
**WAIS**	**R**	FSIQ	Full-Scale IQ

		Verbal IQ	Verbal IQ
		Performance IQ	Performance IQ
		Working Memory	-
		Processing Speed	-
**WAIS**	**IV**	FSIQ	Full-Scale IQ
		Verbal IQ	Verbal Comprehension Index
		Performance IQ	Perceptual Reasoning Index
		Working Memory	Working Memory Index
		Processing Speed	Processing Speed Index
**WAIS**	**III**	FSIQ	Full-Scale IQ
		Verbal IQ	Verbal Comprehension Index [a]
		Performance IQ	Perceptual Organisation Index [b]
		Working Memory	Working Memory Index
		Processing Speed	Processing Speed Index
**WASI**	**-**	FSIQ	Full-Scale IQ
		Verbal IQ	Verbal IQ
		Performance IQ	Performance IQ
		Working Memory	-
		Processing Speed	-

[a] If Verbal Comprehension Index was not available, then Verbal IQ was used.
[b] If Perceptual Organisation Index was not available, then Performance IQ was used.
[c] If Working Memory Index was not available, then Freedom from Distractibility Index was used.

Supplementary Table 2. Available neuropsychology assessments, both before and after surgery (*N* = 500).

**Number and type of assessment(s)**	***N***
At least one preoperative assessment	453
Multiple preoperative assessments	144
At least one postoperative assessment	361
Multiple postoperative assessments	116
At least one preoperative and one postoperative assessment	314

Supplementary Table 3. Demographic information and clinical characteristics (*N* = 500).

**Sex** *N* (% of total sample)	
Males	252 (50)
Females	248 (50)
**Ethnicity** *N* (% of total sample)	
Asian	28 (6)
Black	22 (4)
Mixed	10 (2)
White	390 (78)
Other	7 (1)
Ethnicity not asked or given	43 (9)
**Handedness** *N* (% of total sample)	
Right	348 (70)
Left	100 (20)
No clear hand preference/Ambidextrous	14 (3)
Missing data	38 (8)
**Educational status** *N* (% of total sample)	
Mainstream school	191 (38)
Mainstream school with support	157 (31)
SEN school	59 (12)
Home-schooled/Residential home	13 (3)
Not of school age	52 (10)
Missing data	28 (6)
**Epilepsy characteristics** years, Md [IQR] (range)	
Age at first seizure [a]	3.0 [1.0, 6.0] (0-15.3)
Age of epilepsy onset	3.2 [1.2, 6.5] (0-15.3)
Age at surgery [b]	11.9 [7.8, 15.0] (0.2-19.5)
Duration of epilepsy at time of surgery	6.1 [3.9, 9.6] (0.1-18.4)
**Antiseizure medication (ASM)** *N* (% of total sample)	
**Number of ASMs at preoperative evaluation**	
0	6 (1)
1	111 (22)
2	208 (42)
3	124 (25)
4+	40 (8)
Missing data	11 (2)
**Total number of ASMs trialled (from epilepsy onset to surgery)** [c]	
0	1 (<1)
1-2	82 (16)
3-4	176 (35)

5-6	126 (25)
7-8	61 (12)
9+	31 (6)
Missing data	23 (5)
**Preoperative MRI findings** *N* (% of total sample)	
**MRI laterality**	
Left	240 (48)
Right	202 (40)
Bilateral	38 (8)
Negative	18 (4)
Not applicable	1 (<1)
Missing data	1 (<1)
**MRI status**	
Focal	324 (65)
Diffuse	117 (23)
Multifocal	40 (8)
Negative	18 (4)
Missing data	1 (<1)
**Genetic findings** *N* (% of total sample)	
Pathogenic/likely pathogenic SNV	11 (2)
CNV	2 (<1)
Benign/likely benign SNV	10 (2)
No variant identified	34 (7)
No test administered	443 (89)
**Surgery information** *N* (% of total sample)	
**Type of surgery**	
Lesionectomy	208 (42)
Lobectomy	174 (35)
Hemispherotomy	80 (16)
Disconnection	16 (3)
Corpus callosotomy	11 (2)
Lobectomy + Disconnection	7 (1)
Lesionectomy + Lobectomy	4 (<1)
**Side operated on**	
Left	227 (45)
Right	261 (52)
Not applicable [d]	12 (2)
**Lobe operated on**	
Temporal	236 (47)
Frontal	90 (18)
Multilobar	37 (7)
Parietal	33 (7)
Occipital	10 (2)

Insular	2 (<1)
Not applicable [e]	92 (18)
**Histopathology diagnosis**	
LEAT	126 (25)
MTS	75 (15)
FCD-II	71 (14)
Scarring	40 (8)
NSC	35 (7)
MCD-Other	25 (5)
Rasmussen encephalitis	23 (5)
Vascular	18 (4)
Tuberous sclerosis	11 (2)
N-LEAT	8 (2)
Normal result	7 (1)
FCD-NOS	6 (1)
M-MCD	2 (<1)
Histopathology not collected or report not available	53 (11)

Abbreviations: ASM = antiseizure medication; CNV = copy number variation; DNET = dysembryoplastic neuroepithelial tumour; FCD-II = focal cortical dysplasia type-II; FCD-NOS = focal cortical dysplasia not otherwise specified; IQR = interquartile range; LEAT = low-grade epilepsy-associated tumour; MCD-Other = malformation of cortical development-other; Md = median; M-MCD = mild malformation of cortical development; MTS = mesial temporal sclerosis; NA = not applicable; N-LEAT = non-low-grade epilepsy-associated tumour; NSC = non-specific epilepsy-associated changes; SD = standard deviation; SEN = special educational needs; SNV = single nucleotide variation.

[a] Age at first seizure and Age of epilepsy onset were kept distinct to account for early, isolated occurrences of febrile seizures.

[b] Three patients were 19 years of age at time of surgery.

[c] One patient had not been trialled on any ASMs prior to surgery due to parental preference. A small proportion of patients (N = 32 patients; 6% of the cohort) had been trialled on one ASM prior to surgery; however, they all had a clearly defined lesion on their preoperative MRI scan and the clinical team recommended they proceed to surgery due to the high likelihood of drug-resistance.

[d] Not applicable was assigned to focal resections that involved the removal of a hypothalamic hamartoma.

[e] Not applicable was assigned to hemispherotomy as well as focal resections that involved the removal of a hypothalamic hamartoma.

Supplementary Table 4. Past and current seizure information (N = 500).

**Family history of epilepsy** *N* (% of total sample)	
Yes	144 (29)
No	322 (64)
Missing data	34 (7)
**Seizure history** *N* (% of total sample)	
**History of infantile spasms**	
Yes	33 (7)
No	417 (83)
Missing data	50 (10)
**History of status epilepticus**	
EPC	17 (3)
Non-convulsive	7 (1)
Convulsive	63 (13)
Mixed	6 (1)
None	358 (72)
Missing data	49 (10)
**History of generalised tonic-clonic seizures**	
Yes	205 (41)
No	241 (48)
Missing data	54 (11)
**Seizures at time of preoperative evaluation** *N* (% of total sample)	
**Spasms**	
Yes	37 (7)
No	416 (83)
Missing data	47 (9)
**Number of seizure types**	
0	5 (1)
1	219 (44)
2	154 (31)
3	50 (10)
4	16 (3)
5	1 (<1)
6	2 (<1)
Missing data	53 (11)

Abbreviations: EPC = epilepsia partialis continua.

Supplementary Table 5. Comparison between cohorts with a single versus multiple preoperative assessments, a single versus multiple postoperative assessments, as well as surgical patients with and without neuropsychology assessment.

	**One versus multiple preoperative assessments**	**One versus multiple postoperative assessments**	**Surgical cohort with versus without neuropsychological assessment**
Sex	ns	ns	ns
Handedness	ns	ns	*** The surgery cohort without neuropsychology was more likely to have no hand preference or be left-handed
Educational status	ns	ns	*** The surgery cohort without neuropsychology was more likely to not be of school age or attend a special educational needs school
Preoperative FSIQ	ns	ns	-
Family history of epilepsy	ns	ns	ns
Genetic diagnosis of epilepsy	ns	ns	*** The surgery cohort without neuropsychology was more likely to have a genetic diagnosis of epilepsy
Age of epilepsy onset	ns	ns	*** The surgery cohort without neuropsychology had a younger age of epilepsy onset; $Md_{\text{surgery only}} = 0.4$, $IQR_{\text{surgery only}} = (0.1, 1.2)$ versus $Md_{\text{surgery \& neuropsychology}} = 3.2$, $IQR_{\text{surgery \& neuropsychology}} = (1.4, 6.5)$
Age at surgery	** Patients with one assessment had a younger age at surgery; $Md_{\text{single}} = 11.8$, $IQR_{\text{single}} = (7.9, 15.0)$ versus $Md_{\text{multi}} = 13.4$, $IQR_{\text{multi}} = (10.4, 15.6)$	ns	*** The surgery cohort without neuropsychology had a younger age at surgery; $Md_{\text{surgery only}} = 5.3$, $IQR_{\text{surgery only}} = (2.4, 8.9)$ versus $Md_{\text{surgery \& neuropsychology}} = 11.9$, $IQR_{\text{surgery \& neuropsychology}} = (7.8, 15.0)$
Duration of epilepsy	** Patients with one assessment had	ns	*** The surgery cohort without neuropsychology had

	shorter duration of epilepsy; $Md_{single} = 6.0$, $IQR_{single} = (3.9, 9.7)$ versus $Md_{multi} = 8.2$, $IQR_{multi} = (5.3, 10.8)$		shorter duration of epilepsy; $Md_{surgery\ only} = 3.7$, $IQR_{surgery\ only} = (1.9, 6.8)$ versus $Md_{surgery\ \&\ neuropsychology} = 6.3$, $IQR_{surgery\ \&\ neuropsychology} = (3.9, 9.8)$
History of infantile spasms	ns	ns	*** The surgery cohort without neuropsychology was more likely to have a history of infantile spasms
Spasms at time of preoperative evaluation	ns	ns	*** The surgery cohort without neuropsychology was more likely to have spasms at the time of preoperative evaluation
History of GTC seizures	ns	ns	ns
Number of seizure types at preoperative assessment	ns	ns	ns
Number of ASMs at preoperative assessment	ns	ns	*** The surgery cohort without neuropsychology was receiving a greater number of different ASMs at the time of preoperative evaluation; $M_{surgery\ only} = 2.6$, $SD_{surgery\ only} = 1.0$ versus $M_{surgery\ \&\ neuropsychology} = 2.2$, $SD_{surgery\ \&\ neuropsychology} = 1.0$
Total number of ASMs trialled from epilepsy onset to preoperative evaluation	*** Patients with one assessment had trialled a smaller number of different ASMs over the course of their epilepsy; $M_{single} = 4.4$, $SD_{single} = 2.1$ versus $M_{multi} = 5.5$, $SD_{multi} = 2.6$	ns	*** The surgery cohort without neuropsychology had trialled a larger number of different ASMs over the course of their epilepsy; $M_{surgery\ only} = 5.4$, $SD_{surgery\ only} = 2.6$ versus $M_{neuropsychology} = 4.6$, $SD_{neuropsychology} = 2.4$
MRI laterality	ns	ns	*** The surgery cohort without neuropsychology was more likely to have bilateral MRI abnormalities or be MRI negative

Type of MRI abnormality	* Patients who had multiple assessments were more likely to have non-focal (diffuse or multifocal) MRI abnormalities or be MRI negative	ns	*** The surgery cohort without neuropsychology was more likely to have non-focal (diffuse or multifocal) MRI abnormalities or be MRI negative
Procedure type	ns	ns	*** The surgery cohort without neuropsychology was more likely to have undergone corpus callosotomy or hemispherotomy
Side operated on	ns	ns	ns
Lobe operated on	ns	ns	*** The surgery cohort without neuropsychology was more likely to have undergone corpus callosotomy, hemispherotomy or multilobar surgery
Histopathology	ns	ns	*** The surgery cohort without neuropsychology was more likely to have a diagnosis of MCD-Other, FCD-II, and Scarring
Seizure outcome	ns	ns	*** The surgery cohort without neuropsychology was more likely to not be seizure-free
Postoperative ASM status	ns	ns	ns

p-values were obtained following correction for multiple comparison using the Holm method, which was applied sequentially to each column.

Abbreviations: ASM = antiseizure medication; FCD-II = focal cortical dysplasia type II; FSIQ = Full-Scale IQ; GTC = generalised tonic-clonic seizures; IQR = interquartile range; M = mean; Md = median; MCD-Other = malformation of cortical development-other; ns = not significant; SD = standard deviation.

*p <0.05

**p <0.01

***p <0.001

Supplementary Table 6. Mixed-effects linear regression analyses: preoperative neuropsychological changes in Rasmussen encephalitis versus non-progressive aetiologies.

Model	β	SE	p
**FSIQ**			
Duration of epilepsy	-1.9	0.3	<0.001 ***
Aetiology: Rasmussen encephalitis	-7.9	5.7	0.166
Duration of epilepsy * Aetiology: Rasmussen encephalitis	-2.7	1.1	0.020 *
**Verbal IQ**			
Duration of epilepsy	-1.6	0.2	<0.001 ***
Aetiology: Rasmussen encephalitis	-8.9	4.9	0.070
Duration of epilepsy * Aetiology: Rasmussen encephalitis	-1.3	1.0	0.193
**Performance IQ**			
Duration of epilepsy	-1.1	0.2	<0.001 ***
Aetiology: Rasmussen encephalitis	-8.1	5.4	0.138
Duration of epilepsy * Aetiology: Rasmussen encephalitis	-1.7	1.2	0.153
**Working Memory**			
Duration of epilepsy	-0.8	0.3	0.001 **
Aetiology: Rasmussen encephalitis	-11.9	6.8	0.080
Duration of epilepsy * Aetiology: Rasmussen encephalitis	-0.3	1.5	0.827
**Processing Speed**			
Duration of epilepsy	-1.4	0.2	<0.001 ***
Aetiology: Rasmussen encephalitis	-0.2	6.1	0.975
Duration of epilepsy * Aetiology: Rasmussen encephalitis	-3.4	1.4	0.017 *
**Reading**			
Duration of epilepsy	-1.0	0.2	<0.001 ***
Aetiology: Rasmussen encephalitis	0.8	6.3	0.904
Duration of epilepsy * Aetiology: Rasmussen encephalitis	-1.9	1.3	0.141
**Spelling**			
Duration of epilepsy	-0.9	0.2	<0.001 ***
Aetiology: Rasmussen encephalitis	-2.9	6.4	0.655
Duration of epilepsy * Aetiology: Rasmussen encephalitis	-0.4	1.3	0.759
**Numeracy**			
Duration of epilepsy	-1.6	0.3	<0.001 ***
Aetiology: Rasmussen encephalitis	-16.1	6.7	0.017 *
Duration of epilepsy * Aetiology: Rasmussen encephalitis	1.3	1.4	0.367

Abbreviations: FSIQ = Full-Scale IQ; SE = standard error.

*p <0.05
**p <0.01
***p <0.001

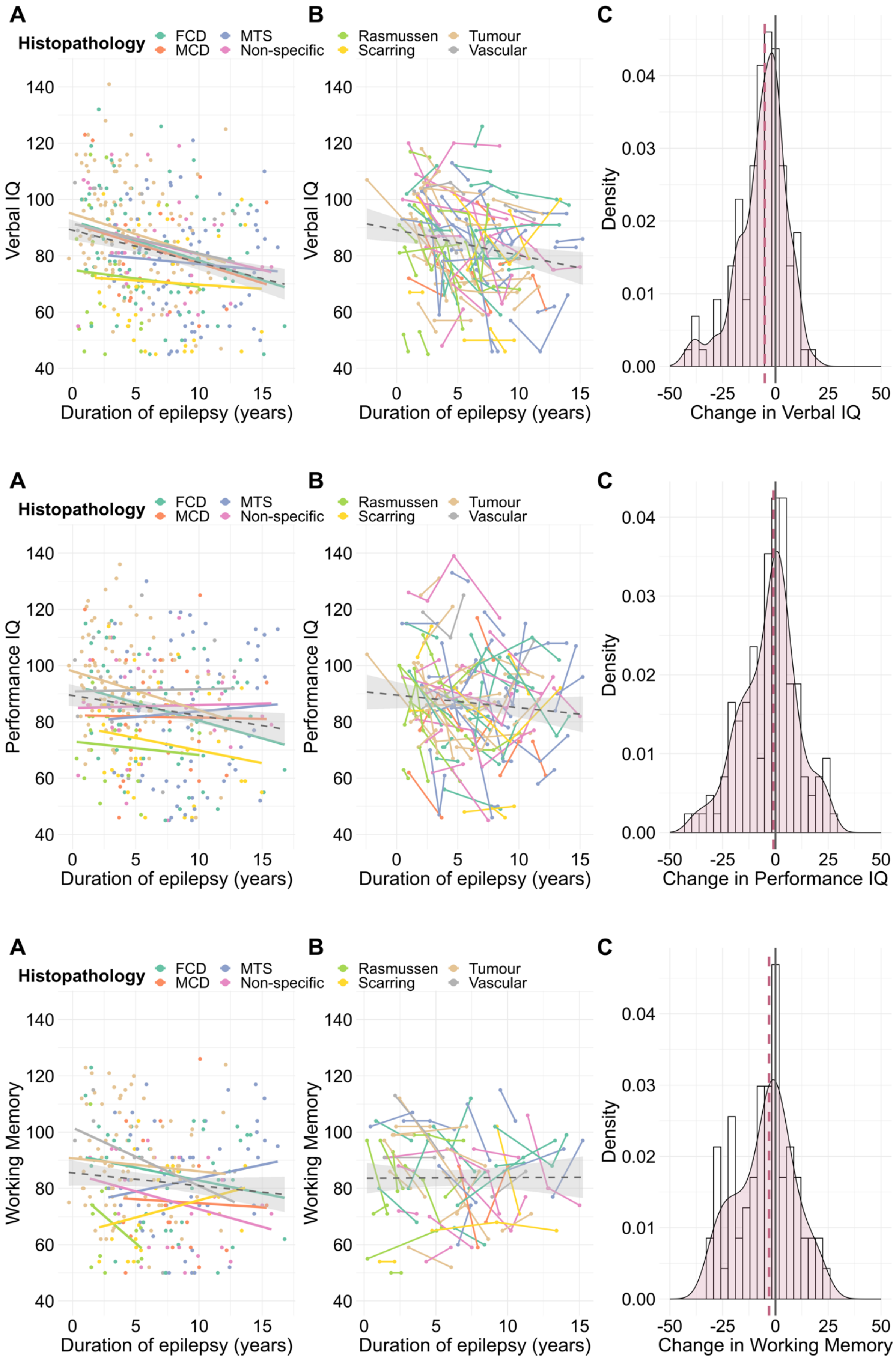
A
B
C
Histopathology
FCD
MCD
MTS
Non-specific
Rasmussen
Scarring
Tumour
Vascular
Verbal IQ
Duration of epilepsy (years)
Density
Change in Verbal IQ
Performance IQ
Change in Performance IQ
Working Memory
Change in Working Memory

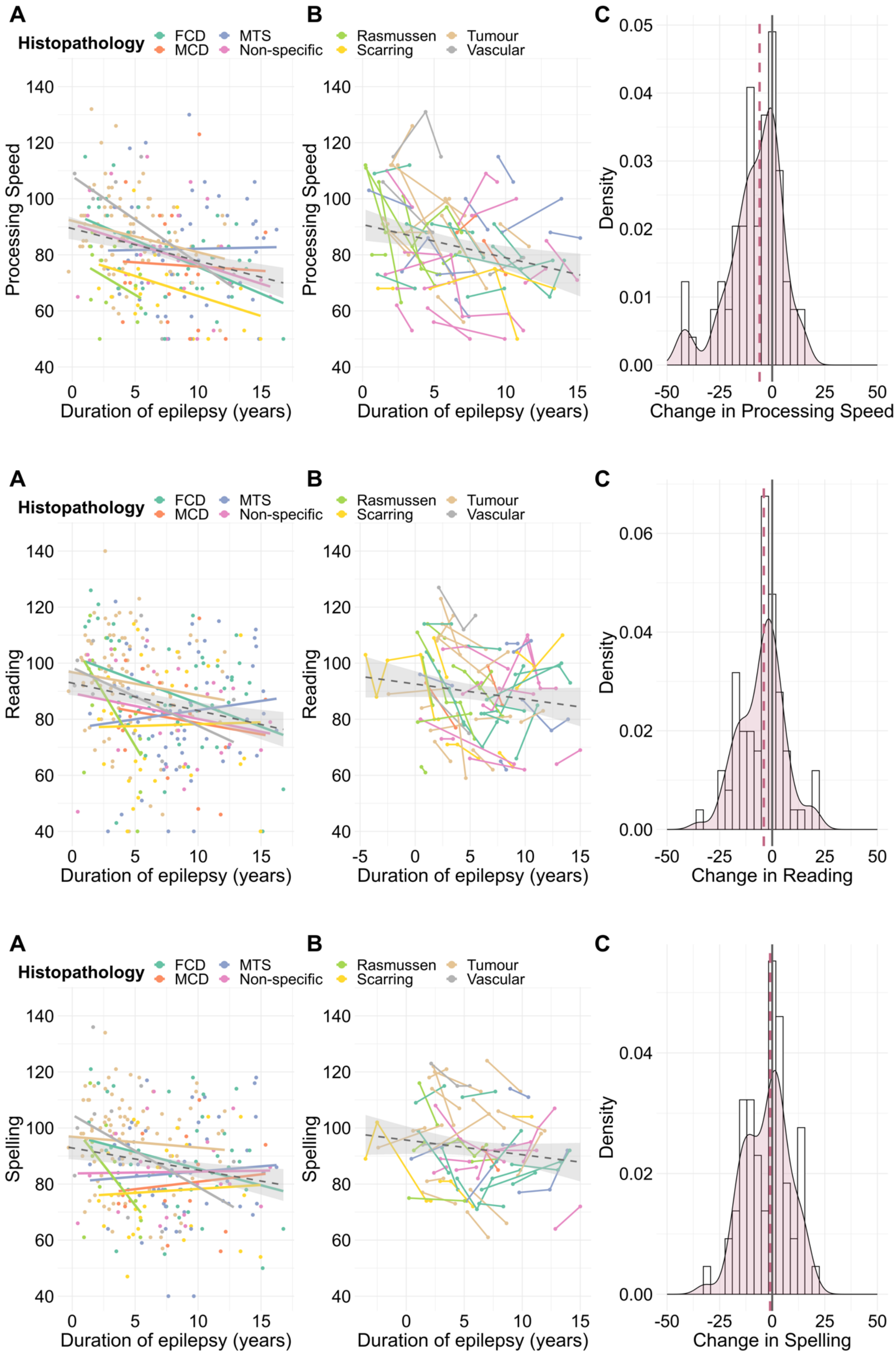
A
B
C
Histopathology
FCD
MCD
MTS
Non-specific
Rasmussen
Scarring
Tumour
Vascular
Processing Speed
Duration of epilepsy (years)
Density
Change in Processing Speed
Reading
Change in Reading
Spelling
Change in Spelling

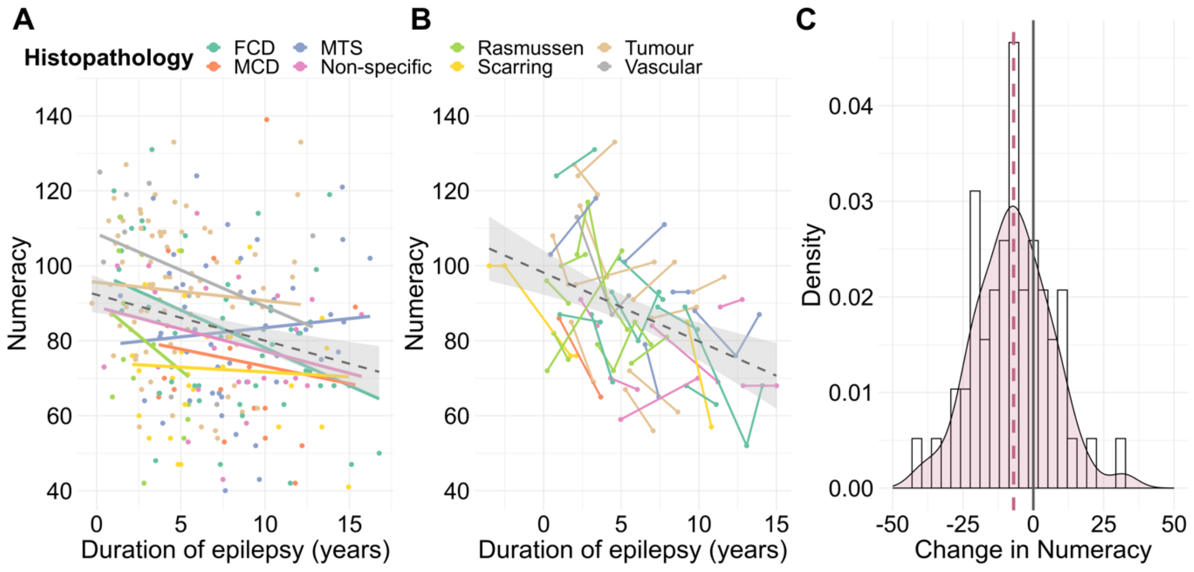

Supplementary Fig. 1. Preoperative changes in neuropsychological performance (A) Cross-sectional preoperative trajectories in Verbal IQ, Performance IQ, Working Memory, Processing Speed, Reading, Spelling, and Numeracy across different aetiologies. A single preoperative assessment was included for each patient. If patients had more than one preoperative assessment, the assessment closest to surgery was chosen. Linear modelling was fitted to all data points (dotted lines); shaded areas represent the 95% confidence intervals. **(B)** Longitudinal preoperative trajectories in Verbal IQ, Performance IQ, Working Memory, Processing Speed, Reading, Spelling, and Numeracy across different aetiologies. Only patients with two or more preoperative assessments were included in the visualisation. All preoperative assessments were included for each patient. Linear modelling was fitted to all data points (dotted lines); shaded areas represent the 95% confidence intervals. **(C)** Individual change in preoperative Verbal IQ, Performance IQ, Working Memory, Processing Speed, Reading, Spelling, and Numeracy. The distribution of change scores is represented using a density plot. The change score for each patient was computed by subtracting the patient's first preoperative score from their last preoperative score. The median change score is displayed with a dashed purple line. Only patients with two or more preoperative assessments were included in the visualisation (patients with unknown histopathology result were also included).

Abbreviations: FCD = focal cortical dysplasia; MCD = malformation of cortical development; MTS = mesial temporal sclerosis.

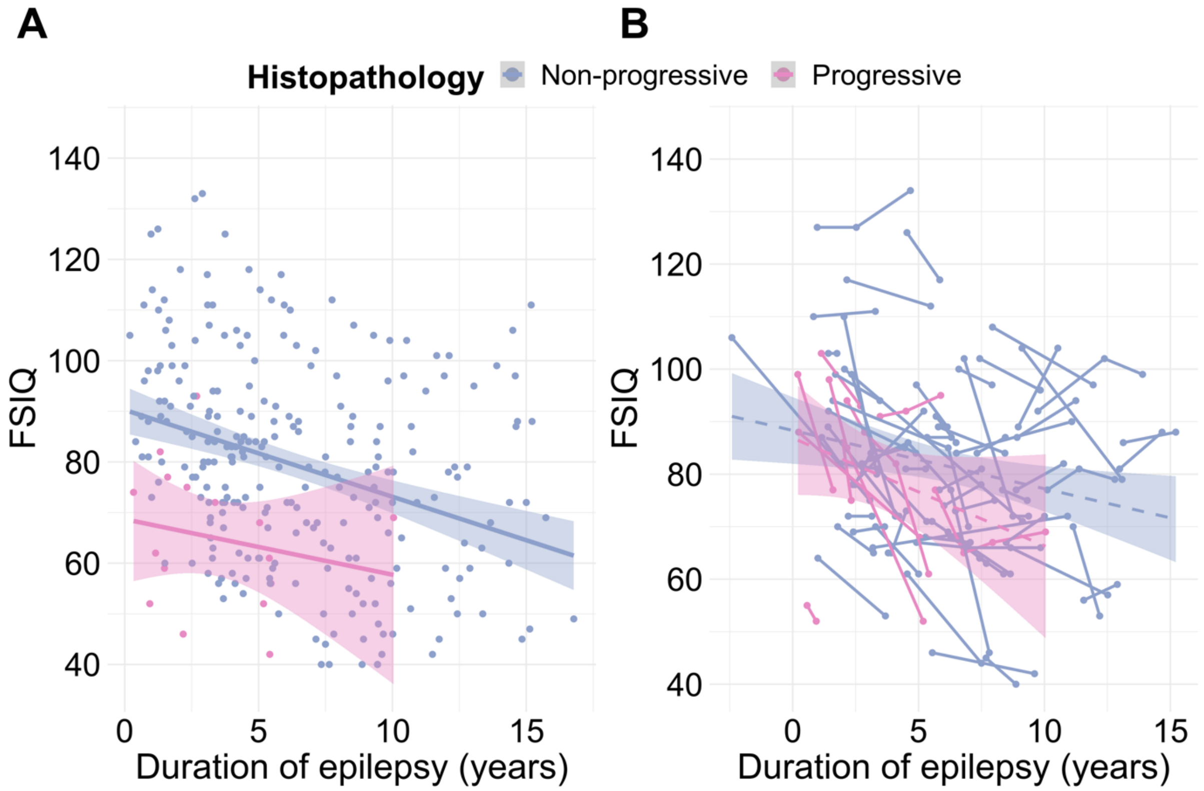
A
B
Histopathology
Non-progressive
Progressive
FSIQ
140
120
100
80
60
40
0
5
10
15
Duration of epilepsy (years)

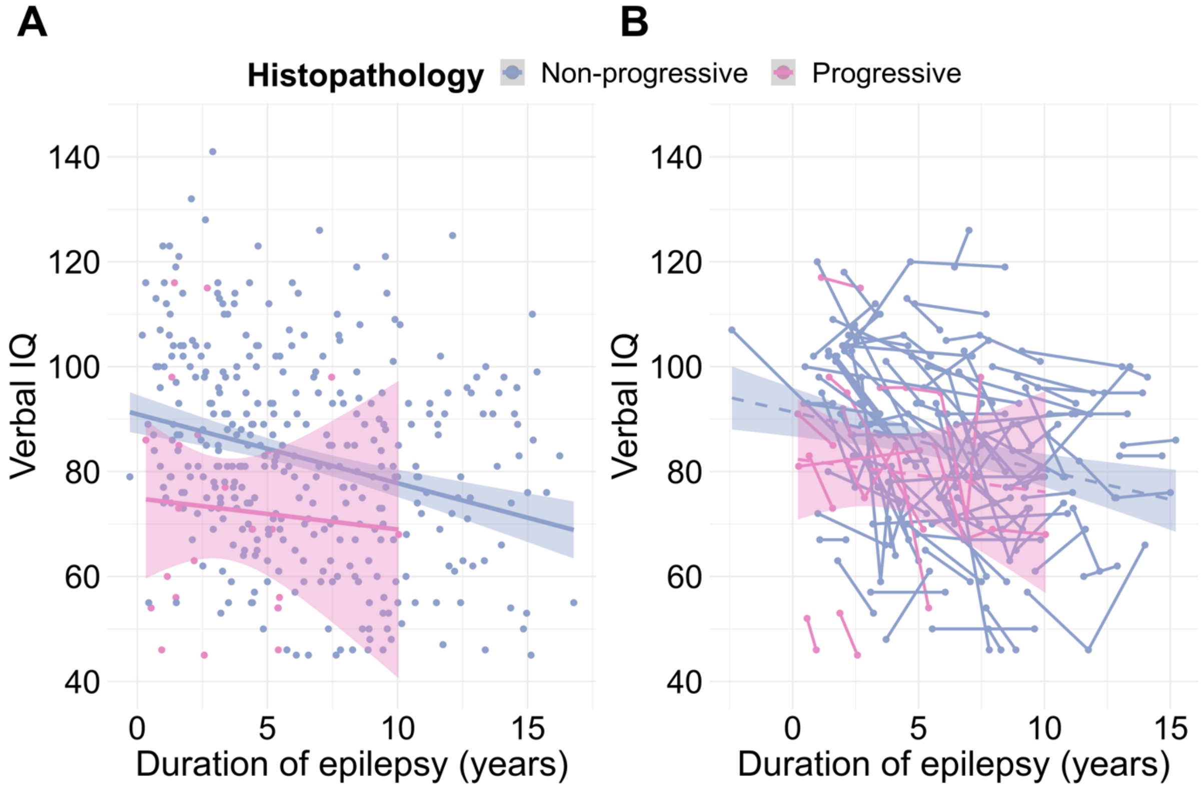
A
B
Histopathology
Non-progressive
Progressive
Verbal IQ
140
120
100
80
60
40
0
5
10
15
Duration of epilepsy (years)

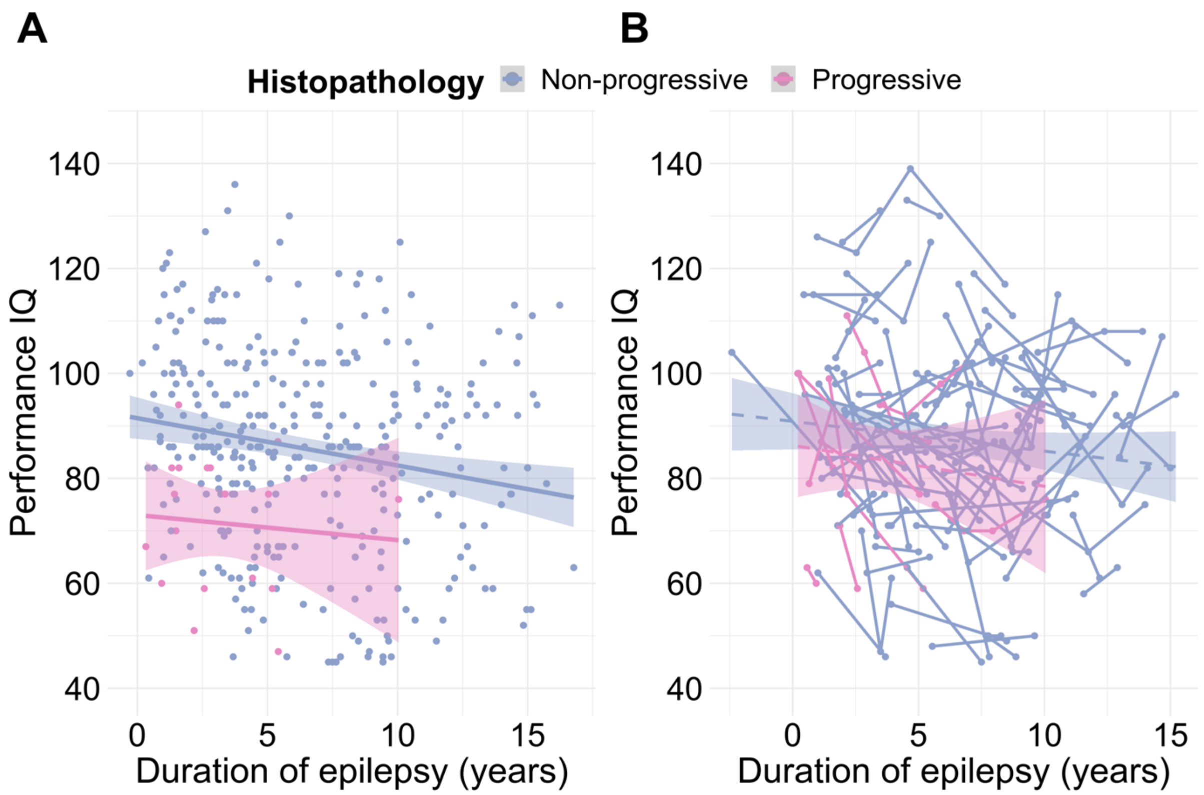

Supplementary Fig. 2. Preoperative changes in neuropsychological performance in progressive versus non-progressive aetiologies (A) Cross-sectional preoperative trajectories in FSIQ, Verbal IQ, and Performance IQ in Rasmussen encephalitis (pink) versus non-progressive aetiologies (blue). A single preoperative assessment was included for each patient. If patients had more than one preoperative assessment, the assessment closest to surgery was chosen. Linear modelling was fitted to all data points, according to aetiology (solid pink lines for Rasmussen encephalitis and solid blue lines for non-progressive aetiologies); shaded areas represent the 95% confidence intervals. **(B)** Longitudinal preoperative trajectories in FSIQ, Verbal IQ, and Performance IQ in Rasmussen encephalitis (pink) versus non-progressive aetiologies (blue). Only patients with two or more preoperative assessments were included in the visualisation. All preoperative assessments were included for each patient. Linear modelling was fitted to all data points, according to aetiology (dotted pink lines for Rasmussen encephalitis and dotted blue lines for non-progressive aetiologies); shaded areas represent the 95% confidence intervals. Working Memory, Processing Speed, Reading, Spelling, and Numeracy were excluded due to small *N*.

Abbreviations: FSIQ = Full-Scale IQ.

Supplementary Table 7. Change in preoperative neuropsychological performance, from first to last preoperative assessment.

	First pre-operative assessment				Last pre-operative assessment				Change score, Md [IQR] (range)	p	Improved *N* (%)	Stable *N* (%)	Declined *N* (%)	Yearly change, M [SD] (range)
	**Age**, years, Md [IQR] (range)	**Duration of epilepsy**, years, Md [IQR] (range)	**Time prior to surgery**, years, Md [IQR] (range)	**Score**, Md [IQR] (range)	**Age**, years, Md [IQR] (range)	**Duration of epilepsy**, years, Md [IQR] (range)	**Time prior to surgery**, years, Md [IQR] (range)	**Score**, Md [IQR] (range)						
**IQ domains**														
FSIQ *N* = 84	9.2 [7.0, 11.8] (3.5, 16.3)	4.6 [2.0, 6.6] (-2.0, 13.1)	2.9 [2.1, 5.0] (0.6, 10.3)	83 [69, 95] (45, 127)	12.4 [9.0, 14.5] (4.4, 18.2)	7.0 [4.5, 9.7] (0.9, 15.2)	0.9 [0.5, 1.4] (0.0, 5.2)	75 [64, 87] (40, 134)	-5 [-11, 2] (-46, 15)	0.021 *	3 (4%)	56 (67%)	25 (30%)	-3 [7] (-33, 11)
Verbal IQ *N* = 126	9.2 [6.9, 11.5] (3.9, 16.3)	4.0 [2.0, 6.6] (-2.4, 13.1)	3.1 [2.2, 5.2] (0.6, 10.4)	87 [74, 99] (48, 120)	12.4 [9.7, 14.6] (5.1, 18.2)	7.2 [4.5, 99.8] (0.9, 15.2)	0.9 [0.6, 1.4] (0.0, 4.0)	79 [67, 91] (45, 126)	-5 [-12, 1] (-43, 18)	0.003 **	7 (6%)	81 (64%)	38 (30%)	-3 [6] (-23, 12)
Performance IQ *N* = 123	9.3 [7.1, 11.5] (3.9, 16.3)	4.5 [2.0, 6.6] (-2.4, 13.1)	3.1 [2.2, 5.3] (0.6, 10.4)	86 [73, 100] (46, 133)	12.5 [9.9, 14.6] (5.1, 18.2)	7.4 [4.6, 9.9] (0.9, 15.7)	0.9 [0.6, 1.4] (0.0, 4.0)	84 [71, 96] (45, 131)	-1 [-12, 4] (-40, 26)	0.209	18 (15%)	68 (55%)	37 (30%)	-1 [8] (-24, 33)
Working Memory *N* = 68	9.8 [8.8, 12.9] (5.5, 16.3)	4.4 [2.0, 6.8] (0.1, 13.1)	3.2 [2.2, 4.8] (1.0, 10.4)	86 [72, 97] (50, 115)	13.1 [11.4, 15.3] (5.8, 11.4)	7.2 [4.5, 9.6] (1.6, 15.2)	1.0 [0.6, 1.5] (0.0, 3.5)	80 [68, 91] (50, 112)	-3 [-14, 5] (-31, 23)	0.091	9 (13%)	37 (54%)	22 (32%)	-2 [9] (-43, 16)
Processing Speed *N* = 71	9.8 [8.6, 12.5] (5.2, 16.3)	4.3 [1.9, 7.0] (0.1, 13.1)	3.3 [2.3, 4.8] (0.8, 10.4)	88 [73, 97] (50, 117)	13.0 [11.3, 15.2] (6.0, 17.4)	7.1 [4.5, 9.9] (1.6, 15.2)	1.0 [0.6, 1.6] (0.0, 3.5)	78 [69, 88] (49, 126)	-6 [-14, 0] (-43, 15)	0.007 **	4 (6%)	40 (56%)	27 (38%)	-4 [6] (-28, 9)
**Academic attainment**														
Reading *N* = 73	9.3 [7.8, 11.2] (4.0, 15.4)	3.9 [2.0, 6.6] (-4.5, 12.9)	3.9 [2.5, 5.5] (0.6, 10.5)	91 [79, 104] (40, 127)	12.5 [10.8, 14.6] (5.9, 16.9)	7.2 [4.4, 9.8] (0.9, 15.7)	1.1 [0.7, 1.5] (0.0, 5.4)	85 [73, 98] (40, 117)	-4 [-13, 1] (-35, 20)	0.130	4 (5%)	46 (63%)	23 (32%)	-2 [6] (-32, 10)
Spelling *N* = 63	9.7 [8.1, 11.3]	4.3 [2.1, 6.6]	3.9 [2.6, 5.4]	89 [81, 100]	13.1 [11.4, 14.8]	7.2 [4.5, 9.9]	1.0 [0.7, 1.4]	92 [77, 100]	-1 [-11, 4] (-32, 19)	0.790	9 (14%)	37 (59%)	17 (27%)	-1 [5] (-23, 9)

	(5.4, 15.4)	(-3.5, 12.9)	(1.0, 10.5)	(47, 125)	(8.0, 16.8)	(1.6, 15.7)	(0.0, 5.4)	(44, 121)						
Numeracy<br>*N* = 56	9.6 [8.1, 12.1]<br>(5.4, 15.4)	4.1 [1.9, 6.5]<br>(-3.5, 12.9)	3.3 [2.5, 5.0]<br>(1.0, 10.5)	89 [75, 98]<br>(53, 127)	12.4 [11.2, 14.7]<br>(8.0, 16.9)	7.1 [3.7, 9.4]<br>(1.6, 15.0)	1.0 [0.6, 1.7]<br>(0.0, 5.4)	80 [65, 92]<br>(42, 133)	-7 [-16, 1]<br>(-40, 32)	0.039 *	6 (11%)	26 (46%)	24 (43%)	-4 [6]<br>(-23, 12)

Abbreviations: IQR = interquartile range; M = mean; Md = median.

*p <0.05

**p <0.01

***p <0.001

Supplementary Table 8. Neuropsychology performance at the time of presurgical evaluation.

	**Score**, Md [IQR] (range)	**<70** *N* (%)	**70-85** *N* (%)	**85-115** *N* (%)	**>115** *N* (%)
**IQ domains**					
FSIQ *N* = 308	75 [61, 89] (40, 133)	115 (37%)	89 (29%)	94 (31%)	10 (3%)
Verbal IQ *N* = 434	80 [67, 93] (45, 141)	129 (30%)	125 (29%)	159 (37%)	21 (5%)
Performance IQ *N* = 410	84 [67, 96] (45, 136)	112 (27%)	95 (23%)	178 (43%)	25 (6%)
Working Memory *N* = 303	80 [65, 94] (50, 126)	96 (32%)	71 (23%)	121 (40%)	15 (5%)
Processing Speed *N* = 307	80 [68, 94] (49, 132)	88 (29%)	96 (31%)	112 (36%)	11 (4%)
**Academic attainment**					
Reading *N* = 335	86 [71, 100] (40, 140)	77 (23%)	78 (23%)	161 (48%)	19 (6%)
Spelling *N* = 310	87 [73, 99] (40, 136)	59 (19%)	84 (27%)	148 (48%)	19 (6%)
Numeracy *N* = 310	82 [66, 97] (40, 139)	103 (33%)	68 (22%)	118 (38%)	21 (7%)

Abbreviations: FSIQ = Full-Scale IQ; IQR = interquartile range; Md = median.

Supplementary Table 9. Change in neuropsychological functioning from pre- to postoperative assessment, using the last preoperative assessment and the first postoperative assessment.

	**Pre-operative assessment score**, Md [IQR] (range)	**Post-operative assessment score**, Md (IQR) (range)	**p**	**Improved** *N* (%)	**Stable** *N* (%)	**Declined** *N* (%)
**IQ domains**						
FSIQ *N* = 157	78 [63, 95] (40, 133)	75 [64, 91] (40, 130)	0.768	24 (15%)	111 (71%)	22 (14%)
Verbal IQ *N* = 286	81 [69, 95] (45, 141)	81 [69, 95] (45, 130)	0.331	32 (11%)	201 (70%)	53 (19%)
Performance IQ *N* = 272	86 [72, 98] (45, 136)	86 [71, 100] (40, 138)	0.948	50 (18%)	173 (64%)	49 (18%)
Working Memory *N* = 191	83 [68, 94] (50, 126)	84 [68, 97] (50, 136)	0.529	44 (23%)	106 (55%)	41 (21%)
Processing Speed *N* = 188	83 [73, 94] (50, 132)	80 [70, 94] (49, 137)	0.515	37 (20%)	108 (57%)	43 (23%)
**Academic attainment**						
Reading *N* = 215	87 [75, 101] (40, 140)	86 [71, 98] (40, 129)	0.128	18 (8%)	149 (69%)	48 (22%)
Spelling *N* = 192	88 [76, 100] (40, 134)	86 [74, 99] (40, 128)	0.465	21 (11%)	133 (69%)	38 (20%)
Numeracy *N* = 189	84 [67, 101] (42, 139)	78 [62, 96] (40, 143)	0.152	33 (17%)	103 (54%)	53 (28%)

Only patients who had both pre- and postoperative assessment were included.

Abbreviations: FSIQ = Full-Scale IQ; IQR = interquartile range; Md = median.

Supplementary Table 10. Change in postoperative neuropsychological performance, from first to last postoperative assessment.

	First postoperative assessment			Last postoperative assessment			Change score, Md [IQR] (range)	p
	**Age**, years, Md [IQR] (range)	**Duration of follow-up**, years, Md [IQR] (range)	**Score**, Md [IQR] (range)	**Age**, years, Md [IQR] (range)	**Duration of follow-up**, years, Md [IQR] (range)	**Score**, Md [IQR] (range)		
**IQ domains**								
FSIQ<br>*N* = 76	13.7 [10.1, 16.5]<br>(3.9, 20.5)	1.3 [1.0, 1.6]<br>(0.0, 8.2)	77 [62, 96]<br>(40, 127)	17.9 [14.9, 23.9]<br>(7.5, 32.9)	7.0 [4.4, 10.1]<br>(1.9, 15.7)	84 [62, 98]<br>(40, 139)	4 [-3, 11]<br>(-16, 22)	0.336
Verbal IQ<br>*N* = 104	12.9 [9.8, 16.3]<br>(3.9, 20.5)	1.2 [1.0, 1.5]<br>(0.0, 5.0)	81 [69, 96]<br>(40, 126)	17.7 [14.5, 22.0]<br>(7.5, 32.9)	6.0 [3.8, 9.4]<br>(1.3, 15.7)	80 [70, 96]<br>(47, 138)	3 [-4, 12]<br>(-23, 28)	0.500
Performance IQ<br>*N* = 102	13.0 [10.0, 16.4]<br>(4.3, 20.5)	1.2 [1.0, 1.6]<br>(0.0, 7.1)	88 [67, 106]<br>(40, 138)	17.9 [14.5, 22.1]<br>(7.5, 32.9)	6.2 [3.9, 9.4]<br>(1.3, 15.7)	92 [71, 104]<br>(45, 135)	2 [-4, 12]<br>(-27, 36)	0.246
Working Memory<br>*N* = 84	13.0 [9.9, 16.4]<br>(6.4, 20.5)	1.3 [1.0, 2.0]<br>(0.2, 5.8)	83 [68, 97]<br>(50, 124)	17.5 [14.5, 21.0]<br>(9.0, 32.9)	5.9 [3.9, 9.2]<br>(1.3, 15.7)	86 [69, 99]<br>(50, 126)	2 [-3, 9]<br>(-37, 25)	0.389
Processing Speed<br>*N* = 85	12.8 [9.4, 16.1]<br>(5.2, 21.4)	1.2 [1.0, 2.0]<br>(0.2, 7.1)	81 [70, 94]<br>(50, 137)	17.2 [13.9, 20.6]<br>(7.5, 32.9)	5.6 [3.8, 9.0]<br>(1.3, 15.7)	84 [64, 97]<br>(49, 124)	0 [-6, 6]<br>(-45, 30)	0.854
**Academic attainment**								
Reading<br>*N* = 89	12.8 [9.9, 16.4]<br>(5.2, 21.8)	1.2 [1.0, 2.0]<br>(0.0, 10.7)	88 [71, 97]<br>(40, 143)	16.8 [14.2, 21.3]<br>(7.5, 31.8)	5.9 [3.8, 9.4]<br>(1.3, 15.4)	84 [69, 105]<br>(40, 125)	0 [-8, 8]<br>(-46, 33)	0.947

Spelling<br>*N* = 80	13.0 [9.9, 16.4]<br>(6.4, 21.8)	1.2 [1.0, 2.0]<br>(0.0, 7.1)	87 [71, 100]<br>(42, 128)	17.1 [14.5, 21.5]<br>(9.0, 31.8)	6.2 [3.9, 9.4]<br>(1.3, 15.4)	92 [69, 103]<br>(40, 130)	2 [-4, 9]<br>(-63, 32)	0.506
Numeracy<br>*N* = 81	13.0 [9.9, 16.4]<br>(6.4, 21.8)	1.3 [1.0, 2.3]<br>(0.0, 10.7)	78 [66, 101]<br>(40, 139)	16.9 [14.2, 21.3]<br>(9.0, 31.8)	6.0 [3.9, 9.4]<br>(1.3, 15.4)	78 [64, 95]<br>(40, 124)	-5 [-15, 3]<br>(-34, 33)	0.262

Abbreviations: FSIQ = Full-Scale IQ; IQR = interquartile range; Md = median.

Supplementary Table 11. Mixed-effects linear regression analyses: postoperative neuropsychological changes in seizure-free versus not seizure-free patients.

Model	β	SE	p
**FSIQ**			
Duration of follow-up	0.0	0.3	0.928
Seizure outcome: Seizure-free	14.1	2.9	<0.001 ***
Duration of follow-up * Seizure outcome: Seizure-free	0.9	0.3	0.004 **
**Verbal IQ**			
Duration of follow-up	-0.1	0.3	0.625
Seizure outcome: Seizure-free	6.9	2.3	0.003 **
Duration of follow-up * Seizure outcome: Seizure-free	1.0	0.3	0.003 **
**Performance IQ**			
Duration of follow-up	0.3	0.3	0.335
Seizure outcome: Seizure-free	10.3	2.6	<0.001 ***
Duration of follow-up * Seizure outcome: Seizure-free	0.7	0.4	0.093
**Working Memory**			
Duration of follow-up	-0.2	0.3	0.544
Seizure outcome: Seizure-free	10.3	2.5	<0.001 ***
Duration of follow-up * Seizure outcome: Seizure-free	1.0	0.4	0.010 *
**Processing Speed**			
Duration of follow-up	-0.6	0.4	0.131
Seizure outcome: Seizure-free	7.5	2.7	0.006 **
Duration of follow-up * Seizure outcome: Seizure-free	0.8	0.5	0.068
**Reading**			
Duration of follow-up	-1.2	0.5	0.015 *
Seizure outcome: Seizure-free	4.6	2.9	0.117
Duration of follow-up * Seizure outcome: Seizure-free	1.6	0.5	0.002 **
**Spelling**			
Duration of follow-up	-0.1	0.5	0.850
Seizure outcome: Seizure-free	7.3	3.0	0.014 *
Duration of follow-up * Seizure outcome: Seizure-free	0.6	0.5	0.220
**Numeracy**			
Duration of follow-up	-0.5	0.5	0.382
Seizure outcome: Seizure-free	13.9	3.2	<0.001 ***
Duration of follow-up * Seizure outcome: Seizure-free	-0.6	0.6	0.268

Abbreviations: SE = standard error.

*p <0.05
**p <0.01
***p <0.001

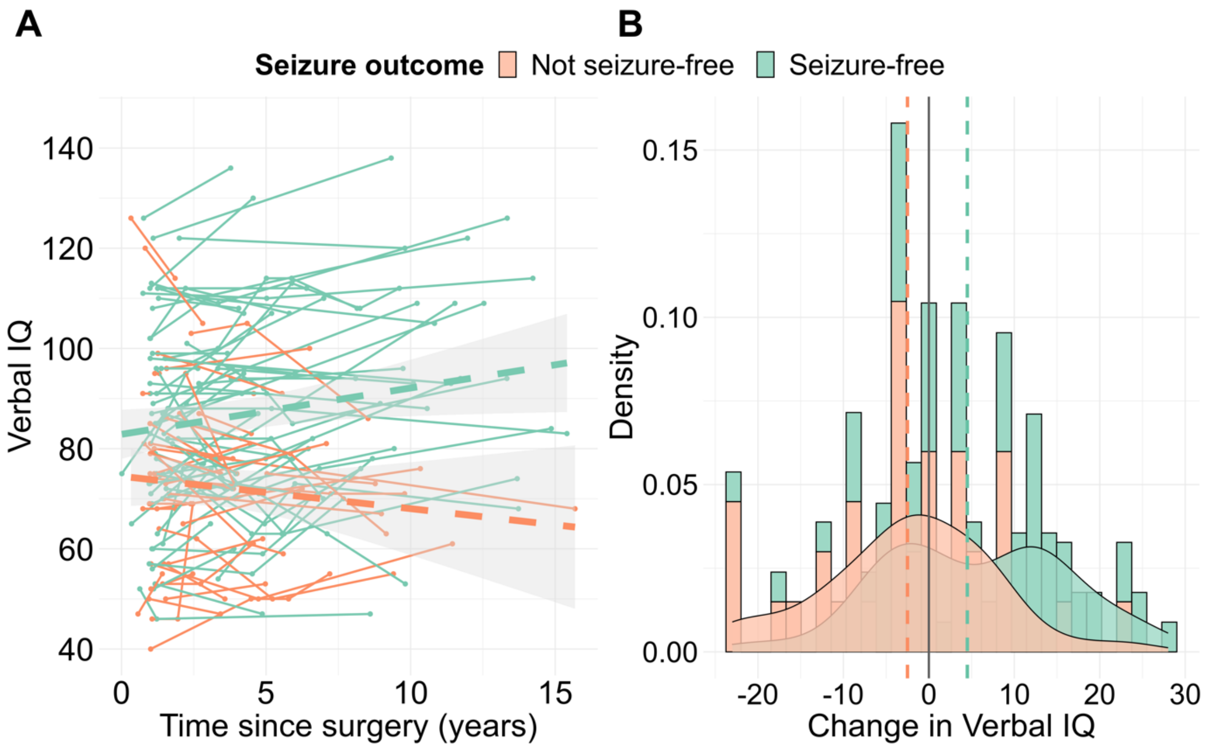
A
B
Seizure outcome
Not seizure-free
Seizure-free
Verbal IQ
140
120
100
80
60
40
0
5
10
15
Time since surgery (years)
Density
0.15
0.10
0.05
0.00
-20
-10
0
10
20
30
Change in Verbal IQ

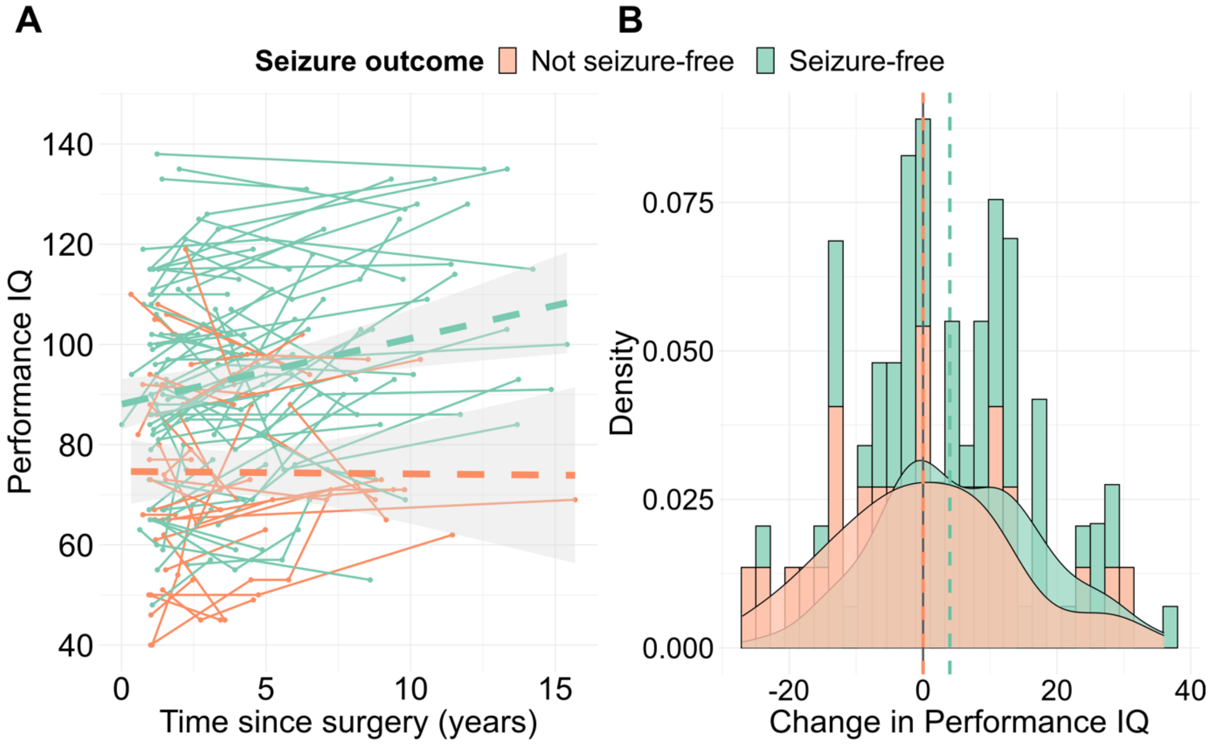
A
B
Seizure outcome
Not seizure-free
Seizure-free
Performance IQ
140
120
100
80
60
40
0
5
10
15
Time since surgery (years)
Density
0.075
0.050
0.025
0.000
-20
0
20
40
Change in Performance IQ

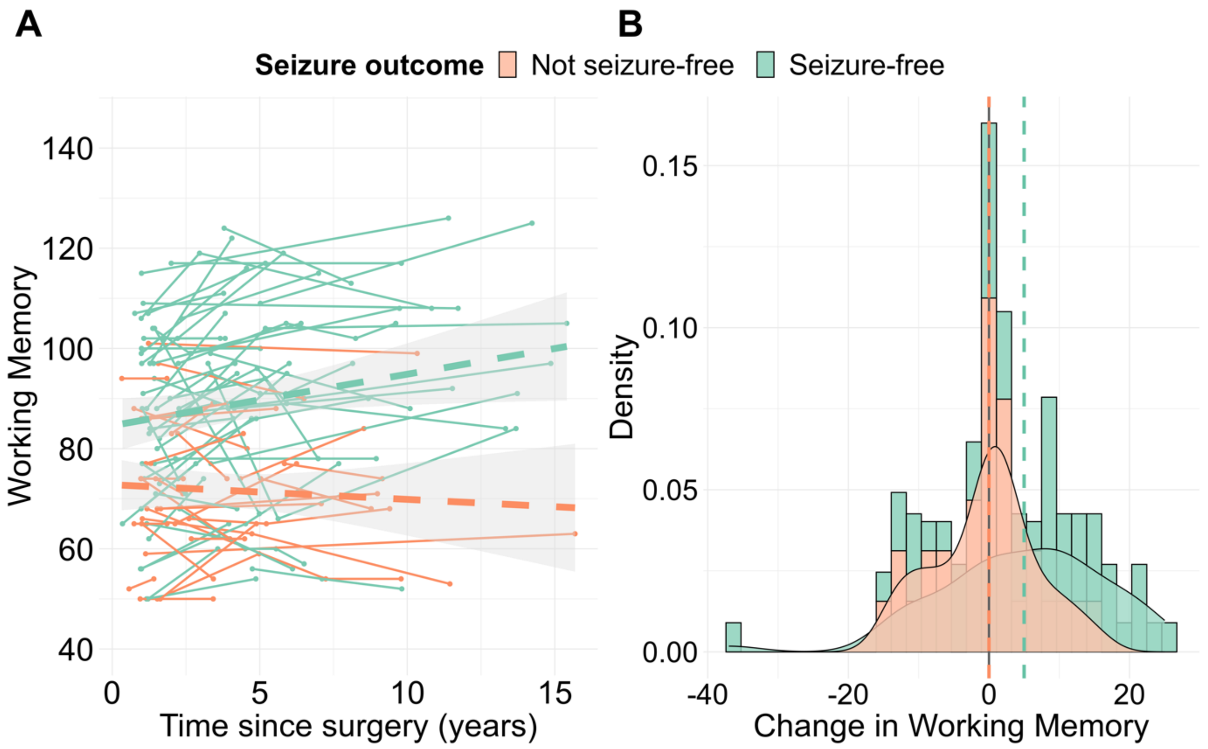

A
B
Seizure outcome
Not seizure-free
Seizure-free
Working Memory
Time since surgery (years)
Density
Change in Working Memory

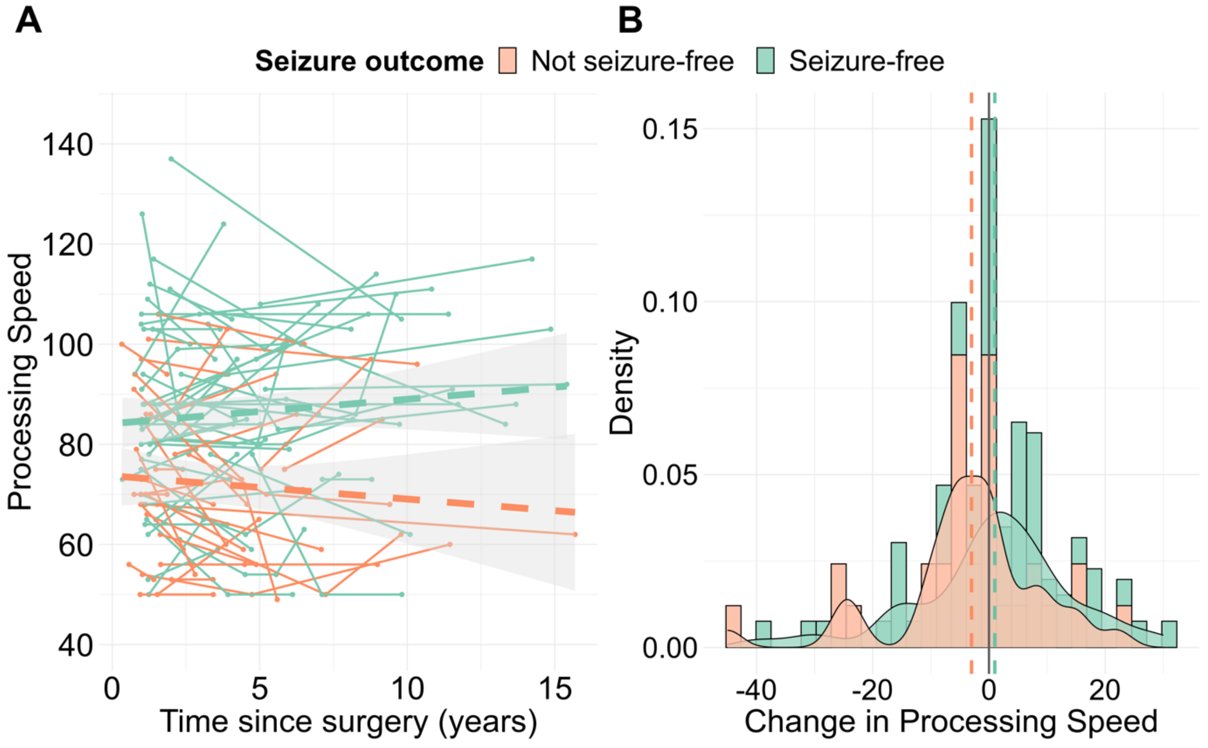

A
B
Seizure outcome
Not seizure-free
Seizure-free
Processing Speed
Time since surgery (years)
Density
Change in Processing Speed

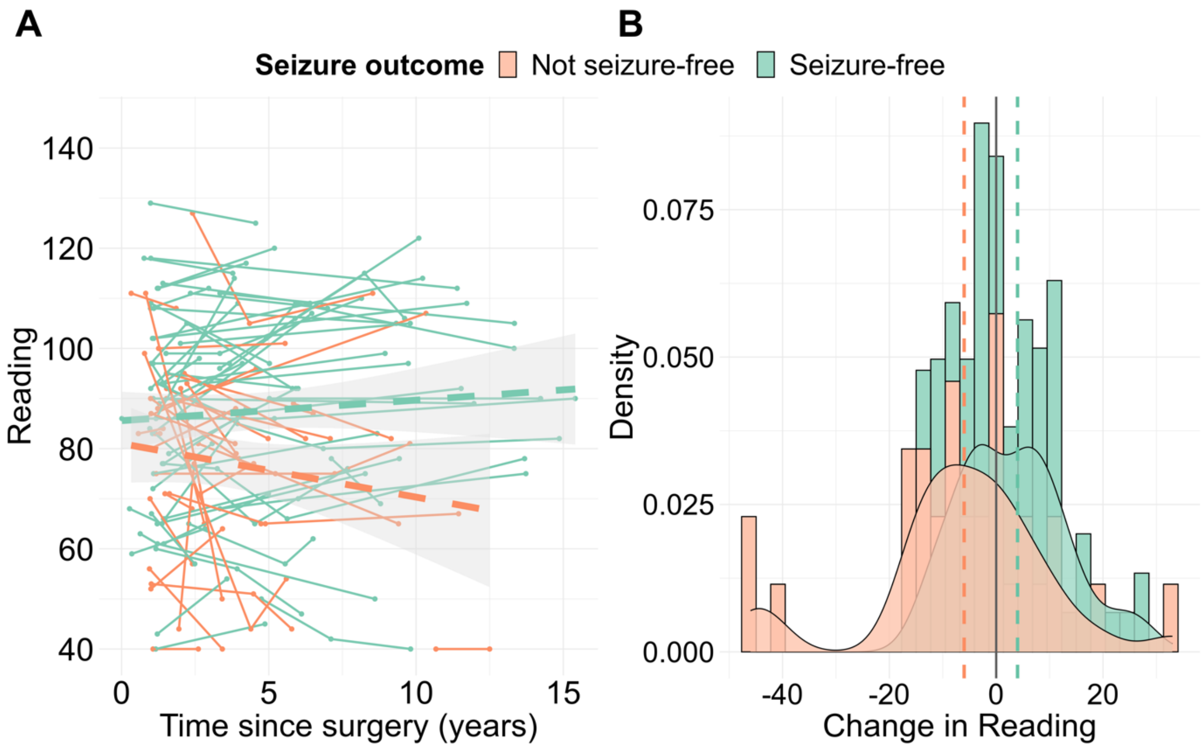
A
B
Seizure outcome
Not seizure-free
Seizure-free
Reading
140
120
100
80
60
40
0
5
10
15
Time since surgery (years)
Density
0.075
0.050
0.025
0.000
-40
-20
0
20
Change in Reading

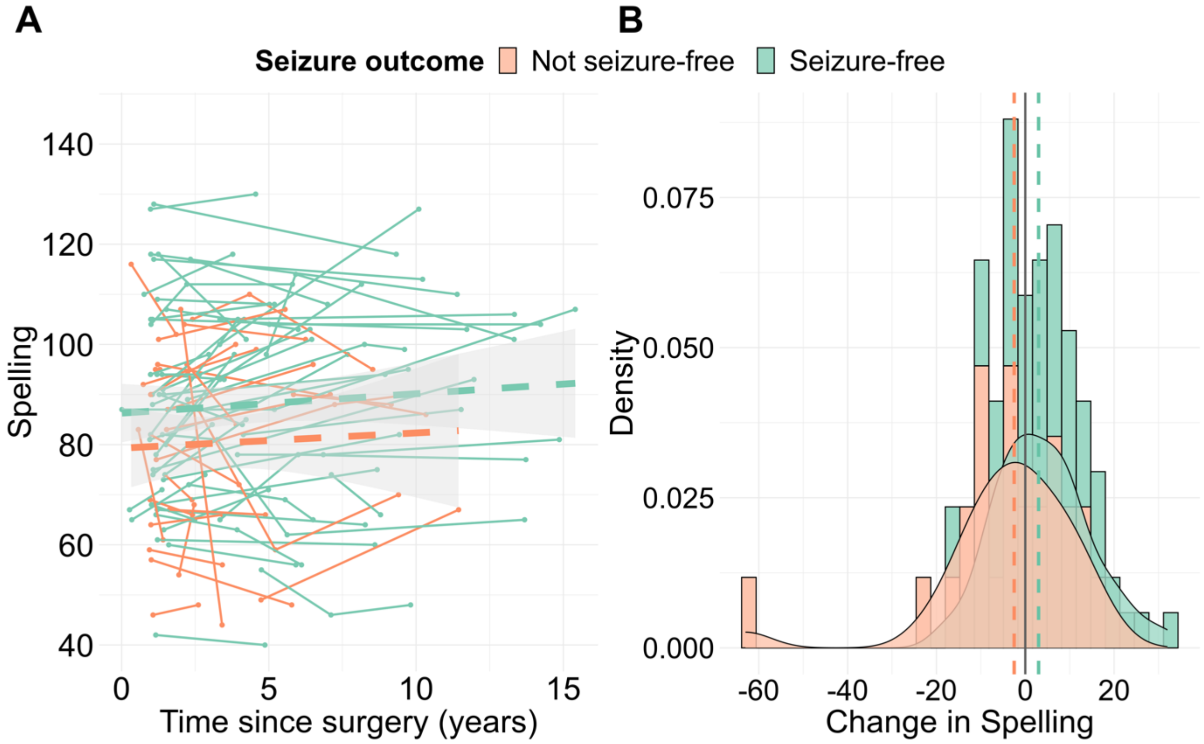
A
B
Seizure outcome
Not seizure-free
Seizure-free
Spelling
140
120
100
80
60
40
0
5
10
15
Time since surgery (years)
Density
0.075
0.050
0.025
0.000
-60
-40
-20
0
20
Change in Spelling

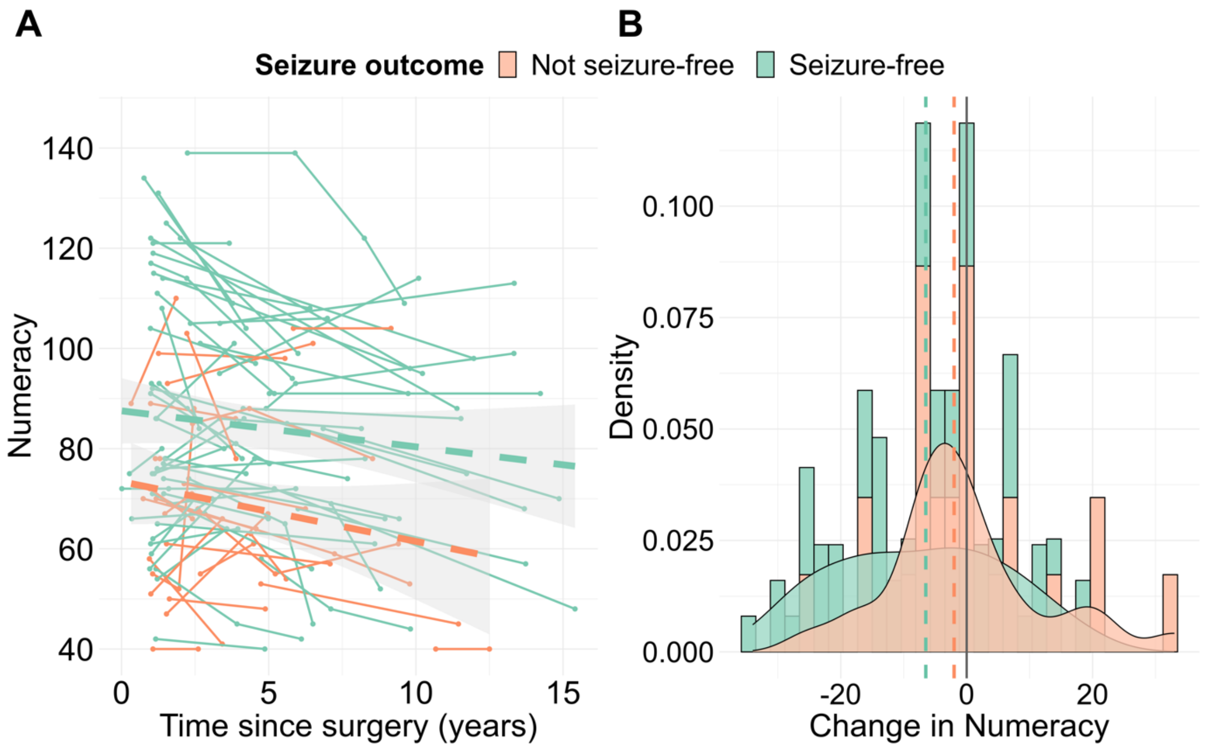

Supplementary Fig. 3. Postoperative changes in neuropsychological performance (A) Longitudinal postoperative trajectories in Verbal IQ, Performance IQ, Working Memory, Processing Speed, Reading, Spelling, and Numeracy in seizure-free (green) versus not seizure-free (orange) patients. Only patients with two or more postoperative assessments were included. All postoperative assessments were included for each patient. Linear modelling was fitted to all data points, according to seizure outcome (dotted green lines for patients who were seizure-free and dotted orange lines for patients who were not seizure-free); shaded areas represent the 95% confidence intervals. **(B)** Individual change in postoperative Verbal IQ, Performance IQ, Working Memory, Processing Speed, Reading, Spelling, and Numeracy in seizure-free (green) versus not seizure-free (orange) patients. The distribution of change scores is represented using a density plot. The change score for each patient was computed by subtracting the patient's first postoperative score from their last postoperative score. The median change scores are displayed with green dashed lines (in seizure-free patients) and with orange dashed lines (in not seizure-free patients). Only patients with two or more postoperative assessments were included in the visualisation.

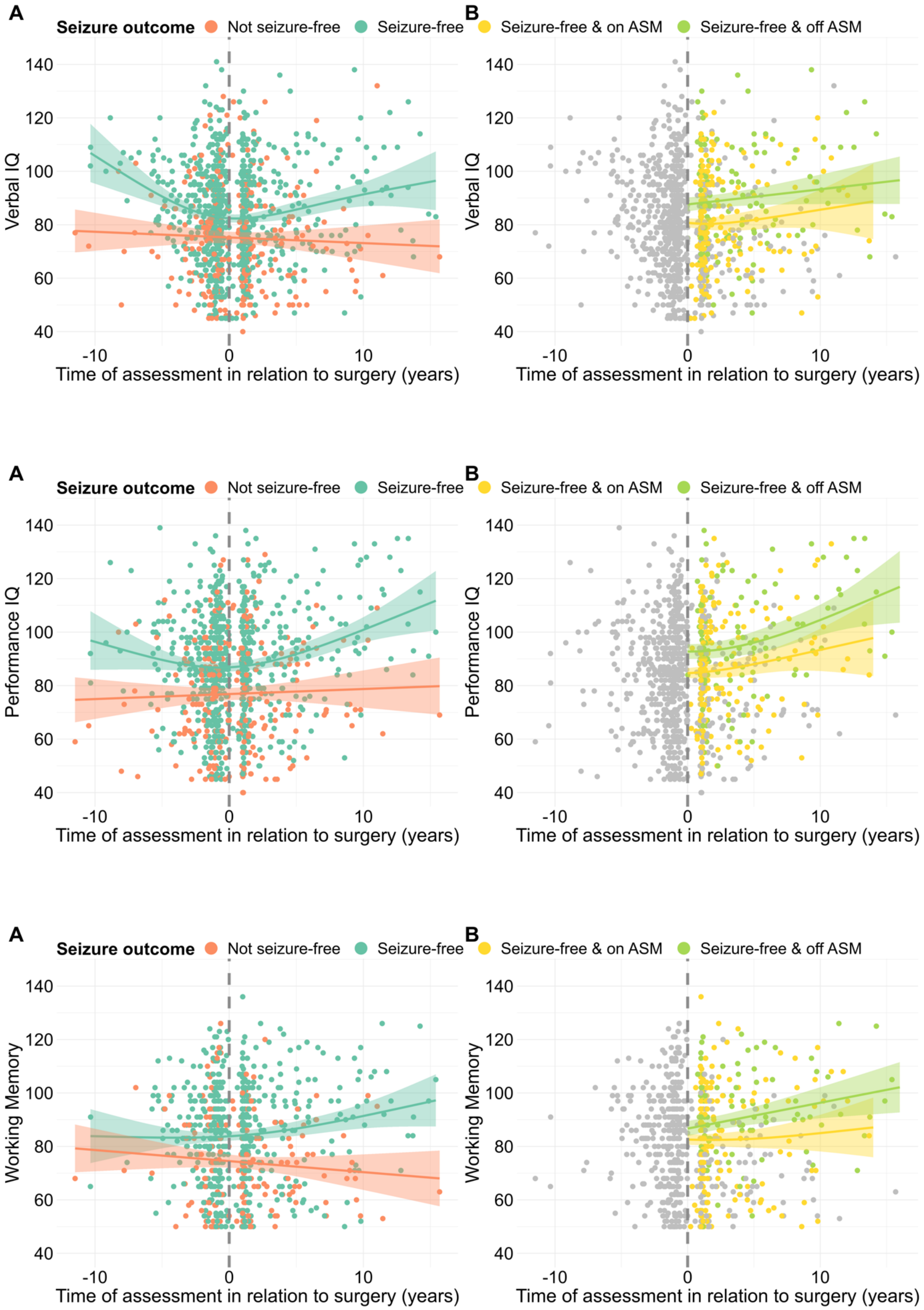
A
Seizure outcome
Not seizure-free
Seizure-free
B
Seizure-free & on ASM
Seizure-free & off ASM
Verbal IQ
Performance IQ
Working Memory
140
120
100
80
60
40
-10
0
10
Time of assessment in relation to surgery (years)

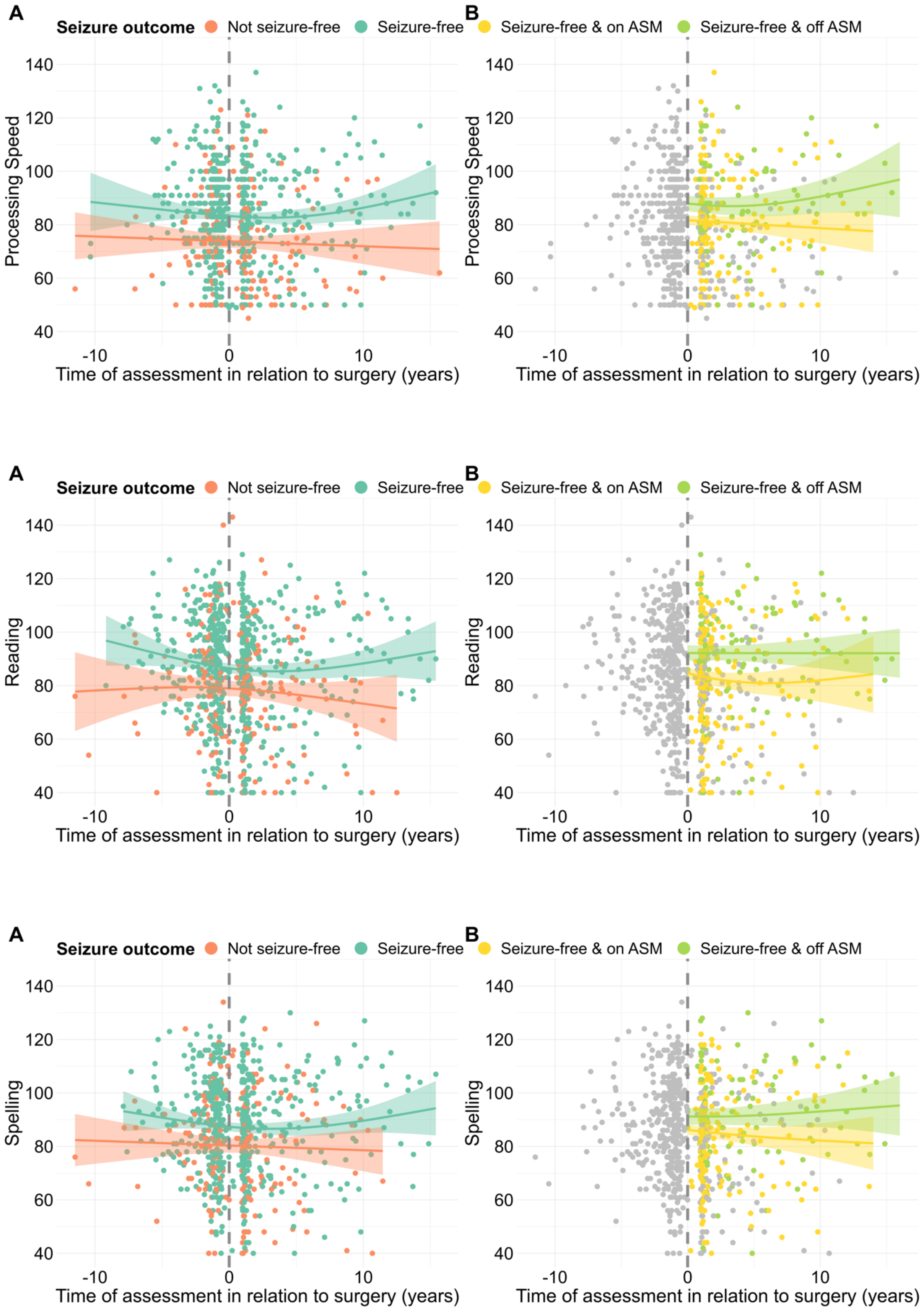
A
Seizure outcome
Not seizure-free
Seizure-free
B
Seizure-free & on ASM
Seizure-free & off ASM
Processing Speed
Reading
Spelling
140
120
100
80
60
40
-10
0
10
Time of assessment in relation to surgery (years)

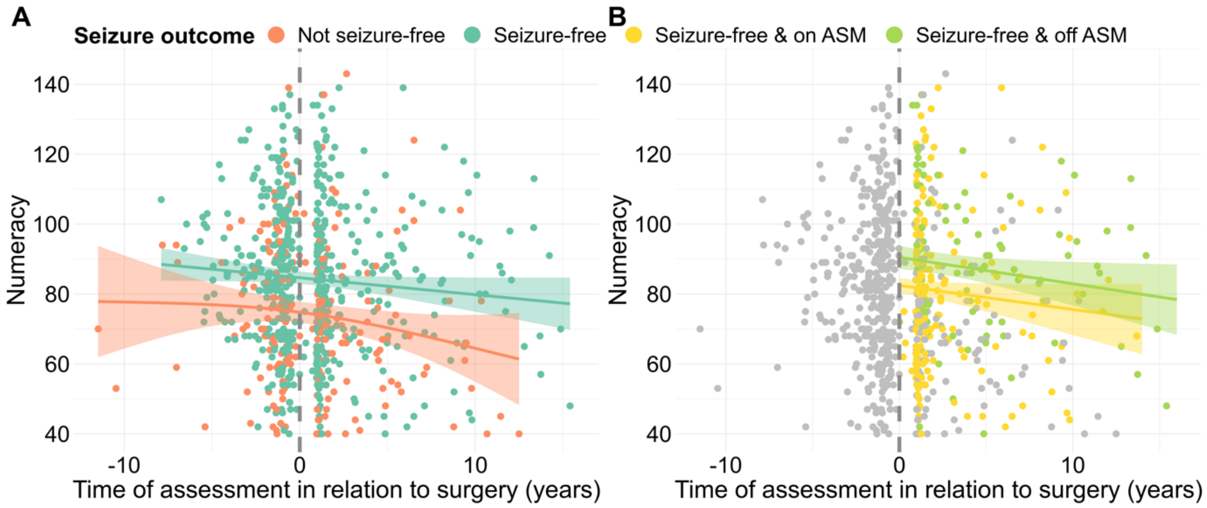

Supplementary Fig. 4. Pre- to postoperative changes in neuropsychological performance **(A)** Pre- and postoperative trajectories in Verbal IQ, Performance IQ, Working Memory, Processing Speed, Reading, Spelling, and Numeracy in seizure-free (green) versus not seizure-free (orange) patients. All patients were included in the visualisation, irrespective of whether they had one or multiple pre- and/or postoperative assessments. If patients had multiple pre- and/or postoperative assessments, all assessments were included for each patient. Generalised additive modelling was fitted to all data points, according to seizure outcome (solid green lines for patients who were seizure-free and solid orange lines for patients who were not seizure-free); shaded areas represent the 95% confidence intervals. **(B)** Pre- and postoperative trajectories in Verbal IQ, Performance IQ, Working Memory, Processing Speed, Reading, Spelling, and Numeracy in seizure-free patients who were still receiving ASMs (yellow) versus no longer receiving ASMs (light green). Grey points represent performance in the preoperative period as well as performance for patients who did not become seizure-free postoperatively. All patients were included in the visualisation, irrespective of whether they had one or multiple pre- and/or postoperative assessments. If patients had multiple pre- and/or postoperative assessments, all assessments were included for each patient. Generalised additive modelling was fitted to all data points, according to postoperative ASM status (solid yellow lines for patients who were still receiving ASMs and solid light green for patients who were no longer receiving ASMs); shaded areas represent the 95% confidence intervals.
Abbreviations: ASM = antiseizure medication.

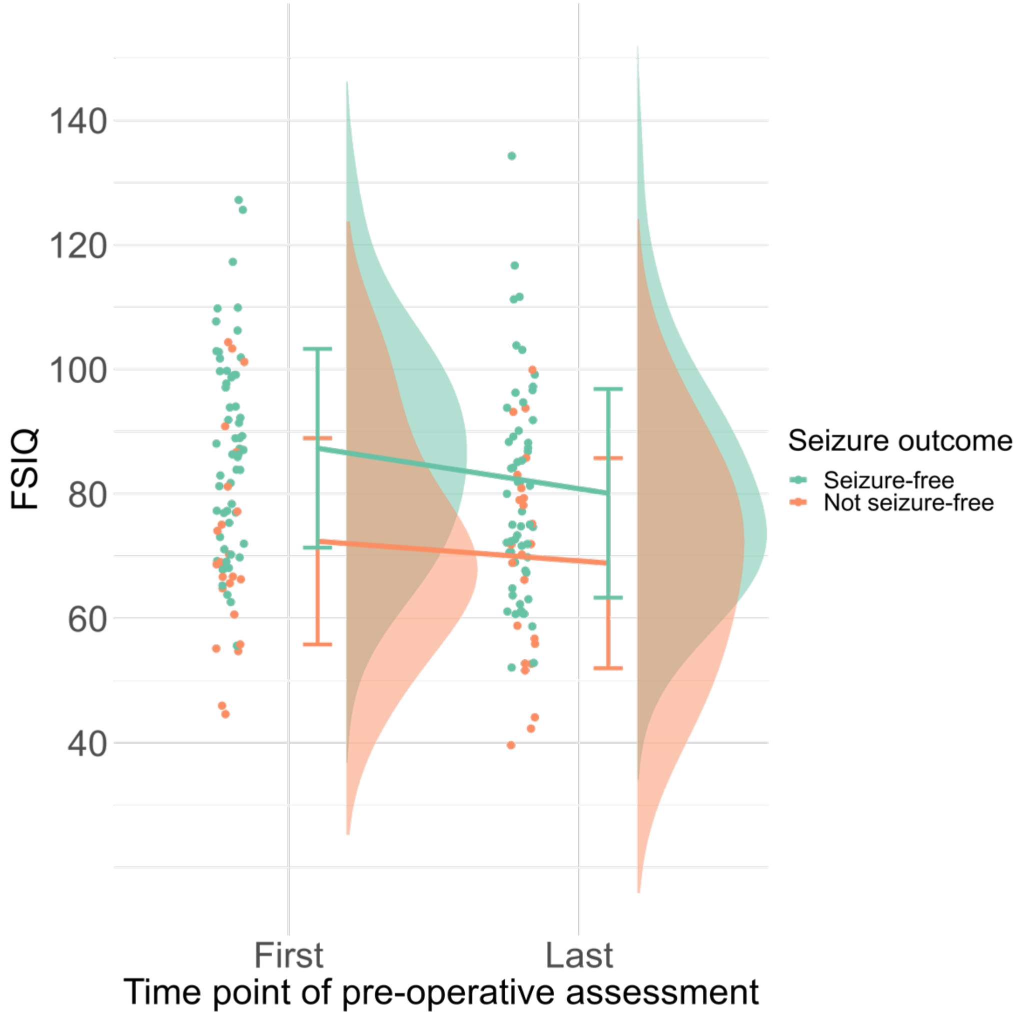

Supplementary Fig. 5. Raincloud plots show the raw data and density functions for FSIQ at first and last preoperative assessment. The lines adjacent to the density functions show the mean and standard error for the groups (connected between the two time points with hashed lines). Only patients with multiple preoperative assessments were included. Patients who became seizure-free postoperatively performed significantly better at both first and last preoperative assessment compared to those patients who did not become seizure-free. Patients also showed higher performance on their first preoperative assessment compared to their last preoperative assessment.

Abbreviations: FSIQ = Full-Scale IQ.

Supplementary Table 12. Two-way ANOVAs investigating the possible interaction effects between time point of preoperative assessment and seizure outcome in patients with multiple preoperative assessments.

	Main effect – time point of preoperative assessment	Main effect – seizure outcome	Interaction effect between time point of preoperative assessment and seizure outcome
**IQ domains**			
FSIQ	p = 0.019	p <0.001	F(1,158) = 0.4, p = 0.511
Verbal IQ	p = 0.007	p <0.001	F(1,234) = 0.3, p = 0.602
Performance IQ	p = 0.276	p <0.001	F(1,228) = 0.7, p = 0.414
Working Memory	p = 0.101	p = 0.278	F(1,222) = 0.0, p = 0.830
Processing Speed	p = 0.011	p <0.001	F(1,128) = 0.2, p = 0.660
**Academic attainment**			
Reading	p = 0.067	p <0.001	F(1,134) = 0.1, p = 0.735
Spelling	p = 0.370	p = 0.135	F(1,116) = 0.0, p = 0.955
Numeracy	p = 0.048	p = 0.200	F(1,102) = 0.1, p = 0.751

Abbreviations: FSIQ = Full-Scale IQ.

Supplementary Table 13. Comparison of seizure-free versus not seizure-free patients, with preoperative neuropsychological data.

Sex	ns
Handedness	ns
Educational status	ns
Preoperative FSIQ	ns
Family history of epilepsy	ns
Genetic diagnosis of epilepsy	ns
Age of epilepsy onset	** Seizure-free patients had an older age of epilepsy onset; $Md_{seizure\text{-}free} = 4.0$, $IQR_{seizure\text{-}free} = (2.0, 7.6)$ versus $Md_{not\ seizure\text{-}free} = 2.9$, $IQR_{not\ seizure\text{-}free} = (2.0, 5.5)$
Age at surgery	ns
Duration of epilepsy	ns
History of infantile spasms	ns
Spasms at time of preoperative evaluation	ns
History of GTC seizures	ns
Number of seizure types at preoperative assessment	ns
Number of ASMs at preoperative assessment	ns
Total number of ASMs trialled from epilepsy onset to preoperative evaluation	** Seizure-free patients had trialled a fewer number of different ASMs over the course of their epilepsy; $M_{seizure\text{-}free} = 4.4$, $SD_{seizure\text{-}free} = 2.2$ versus $M_{not\ seizure\text{-}free} = 5.5$, $SD_{not\ seizure\text{-}free} = 2.5$
MRI laterality	ns
Type of MRI abnormality	*** Patients who were not seizure-free were more likely to have non-focal (diffuse or multifocal) MRI abnormalities or be MRI negative
Procedure type	ns
Side operated on	ns
Lobe operated on	ns
Histopathology	*** Patients who were not seizure-free were more likely to have a diagnosis of MCD-Other, Tuberous sclerosis, Vascular aetiology, and Non-specific epilepsy-associated changes.
Postoperative ASM status	*** Patients who were seizure-free were more likely to be off ASMs postoperatively.

p-values were obtained following correction for multiple comparison using the Holm method.

Abbreviations: ASM = antiseizure medication; FCD-II = focal cortical dysplasia type II; FSIQ = Full-Scale IQ; GTC = generalised tonic-clonic seizures; ns = not significant.

*p <0.05

**p <0.01

***p <0.001

Supplementary Table 14. Relationships between patient characteristics and neuropsychological functioning at different time points.

	Pre-operative trajectory – relative rate of change			Performance at pre-operative assessment			Performance at first post-operative assessment			Performance at last post-operative assessment			Change from pre- to first post-operative assessment			Change from pre- to last post-operative assessment			Post-operative trajectory – relative rate of change		
	**FSIQ**	**VIQ**	**PIQ**	**FSIQ**	**VIQ**	**PIQ**	**FSIQ**	**VIQ**	**PIQ**	**FSIQ**	**VIQ**	**PIQ**	**FSIQ**	**VIQ**	**PIQ**	**FSIQ**	**VIQ**	**PIQ**	**FSIQ**	**VIQ**	**PIQ**
Right-handed (versus Left-handed/ Ambidextrous/ Handedness not established)	-0.13	-0.05	0.03	0.19 *	0.16 *	0.18 *	0.21 *	0.13	0.14	0.13	0.10	0.10	-0.07	0.04	0.05	-0.06	0.05	-0.03	-0.29	-0.03	-0.23
Mainstream school (versus Mainstream school with support /SEN/ Not attending school/ Not of school age)	0.32 *	0.14	0.25 *	0.60 ^{h} ***	0.54 ^{h} ***	0.56 ^{h} ***	0.58 ^{h} ***	0.58 ^{h} ***	0.55 ^{h} ***	0.58 ^{h} ***	0.54 ^{h} ***	0.52 ^{h} ***	0.00	0.00	-0.06	0.00	-0.03	-0.10	-0.06	0.21	-0.23
Initial performance [a]	-0.26 *	-0.19 *	-0.25 **	-	-	-	0.86 ^{h} ***	0.84 ^{h} ***	0.82 ^{h} ***	0.82 ^{h} ***	0.80 ^{h} ***	0.78 ^{h} ***	-0.21 **	-0.34 ^{h} ***	-0.17 **	-0.15	-0.33 ^{h} ***	-0.22 ^{h} ***	0.03	-0.09	-0.21
Family history of epilepsy (versus No family history of epilepsy)	0.08	0.02	-0.06	0.10	0.08	0.09	0.02	-0.04	0.01	0.07	-0.06	0.02	0.06	-0.11	-0.09	-0.02	-0.15 *	-0.10	-0.03	-0.10	0.06
Genetic diagnosis of epilepsy (versus No genetic diagnosis)	*n*	*n*	*n*	-0.21	-0.24	-0.41	-0.08	-0.12	-0.15	-0.22	-0.28	-0.32	-0.06	-0.21	-0.44	-0.15	-0.30	-0.48	*n*	*n*	*n*
Age of epilepsy onset<br>(1) No covariate<br>(2) Controlling for Age at surgery<br>(3) Controlling for Duration of epilepsy	(1) -0.15<br>(2) -0.26 *<br>(3) 0.00	(1) -0.07<br>(2) -0.17<br>(3) 0.07	(1) 0.08<br>(2) -0.01<br>(3) 0.18	(1) 0.26 ^{h} ***<br>(2) 0.30 ^{h} ***<br>(3) 0.13 *	(1) 0.22 ^{h} ***<br>(2) 0.24 ^{h} ***<br>(3) 0.12 *	(1) 0.26 ^{h} ***<br>(2) 0.25 ^{h} ***<br>(3) 0.20 ^{h} ***	(1) 0.30 ^{h} ***<br>(2) 0.26 ^{h} ***<br>(3) 0.27 ^{h} ***	(1) 0.24 ^{h} ***<br>(2) 0.21 ^{h} ***<br>(3) 0.19 ^{h} ***	(1) 0.28 ^{h} ***<br>(2) 0.23 ^{h} ***<br>(3) 0.25 ^{h} ***	(1) 0.29 ^{h} ***<br>(2) 0.23 ^{h} ***<br>(3) 0.27 ^{h} ***	(1) 0.25 ^{h} ***<br>(2) 0.21 ^{h} ***<br>(3) 0.22 ^{h} ***	(1) 0.27 ^{h} ***<br>(2) 0.21 ^{h} ***<br>(3) 0.27 ^{h} ***	(1) 0.04<br>(2) -0.10<br>(3) 0.26 **	(1) 0.04<br>(2) -0.04<br>(3) 0.12	(1) -0.01<br>(2) -0.07<br>(3) 0.09	(1) 0.06<br>(2) -0.07<br>(3) 0.30 ***	(1) 0.05<br>(2) -0.04<br>(3) 0.14 *	(1) -0.01<br>(2) -0.09<br>(3) 0.14 *	(1) 0.18<br>(2) 0.25<br>(3) 0.10	(1) 0.02<br>(2) 0.10<br>(3) -0.06	(1) 0.16<br>(2) 0.12<br>(3) 0.20

	Pre-operative trajectory – relative rate of change			Performance at pre-operative assessment			Performance at first post-operative assessment			Performance at last post-operative assessment			Change from pre- to first post-operative assessment			Change from pre- to last post-operative assessment			Post-operative trajectory – relative rate of change		
	FSIQ	VIQ	PIQ	FSIQ	VIQ	PIQ	FSIQ	VIQ	PIQ	FSIQ	VIQ	PIQ	FSIQ	VIQ	PIQ	FSIQ	VIQ	PIQ	FSIQ	VIQ	PIQ
Age at surgery<br>(1) No covariate<br>(2) Controlling for Age of epilepsy onset<br>(3) Controlling for Duration of epilepsy	(1) 0.18<br>(2) 0.27 *<br>(3) 0.04	(1) 0.19 *<br>(2) 0.24 **<br>(3) 0.10	(1) 0.22 *<br>(2) 0.21 *<br>(3) 0.18	(1) 0.06<br>(2) -0.18 **<br>(3) 0.14 *	(1) -0.03<br>(2) -0.10 *<br>(3) 0.15 **	(1) 0.08<br>(2) 0.00<br>(3) 0.21 h ***	(1) 0.17 *<br>(2) 0.04<br>(3) 0.34 h ***	(1) 0.11 *<br>(2) 0.00<br>(3) 0.27 h ***	(1) 0.18 **<br>(2) 0.08<br>(3) 0.29 h ***	(1) 0.21 **<br>(2) 0.10<br>(3) 0.34 h ***	(1) 0.14 **<br>(2) 0.04<br>(3) 0.28 h ***	(1) 0.22 h ***<br>(2) 0.14 *<br>(3) 0.31 h ***	(1) 0.36 h ***<br>(2) 0.36 h ***<br>(3) 0.23 **	(1) 0.19 **<br>(2) 0.19 **<br>(3) 0.14 *	(1) 0.14 *<br>(2) 0.16 **<br>(3) 0.07	(1) 0.40 h ***<br>(2) 0.39 h ***<br>(3) 0.27 h ***	(1) 0.21 h ***<br>(2) 0.21 h ***<br>(3) 0.16 **	(1) 0.22 h ***<br>(2) 0.23 h ***<br>(3) 0.12 *	(1) -0.18<br>(2) -0.25<br>(3) 0.02	(1) -0.25<br>(2) -0.26<br>(3) -0.18	(1) 0.19<br>(2) 0.15<br>(3) 0.21
Duration of epilepsy [b]<br>(1) No covariate<br>(2) Controlling for Age of epilepsy onset<br>(3) Controlling for Age at surgery	(1) 0.31 **<br>(2) 0.27 *<br>(3) 0.26 *	(1) 0.23 *<br>(2) 0.23 *<br>(3) 0.17	(1) 0.13<br>(2) 0.21 *<br>(3) 0.03	(1) -0.31 h ***<br>(2) -0.23 h ***<br>(3) -0.34 h ***	(1) -0.25 h ***<br>(2) -0.17 h ***<br>(3) -0.29 h ***	(1) -0.17 h ***<br>(2) -0.05<br>(3) -0.25 h ***	(1) -0.16 *<br>(2) -0.06<br>(3) -0.33 h ***	(1) -0.18 **<br>(2) -0.11 *<br>(3) -0.30 h ***	(1) -0.13 *<br>(2) 0.02<br>(3) -0.26 h ***	(1) -0.11<br>(2) 0.00<br>(3) -0.30 h ***	(1) -0.13 *<br>(2) -0.05<br>(3) -0.27 h ***	(1) -0.07<br>(2) 0.08<br>(3) -0.23 h ***	(1) 0.33 h ***<br>(2) 0.41 h ***<br>(3) 0.17 *	(1) 0.13 *<br>(2) 0.17 **<br>(3) 0.05	(1) 0.16 **<br>(2) 0.18 **<br>(3) 0.10	(1) 0.33 h ***<br>(2) 0.43 h ***<br>(3) 0.15	(1) 0.16 **<br>(2) 0.21 h ***<br>(3) 0.05	(1) 0.22 h ***<br>(2) 0.26 h ***<br>(3) 0.14 *	(1) -0.26<br>(2) -0.22<br>(3) -0.20	(1) -0.17<br>(2) -0.18<br>(3) -0.01	(1) 0.04<br>(2) 0.13<br>(3) -0.11
History of infantile spasms (versus No history of infantile spasms)	*n*	*n*	*n*	-0.19	-0.23	-0.24	-0.21	-0.19	-0.21	-0.20	-0.20	-0.21	-0.15	0.00	-0.08	-0.16	-0.02	-0.11	*n*	*n*	*N*
Spasms at time of pre-operative evaluation (versus No spasms at time of pre-operative evaluation)	*n*	*n*	*n*	-0.26	-0.29<br>**	-0.29<br>*	-0.15	-0.15	-0.26	-0.13	-0.15	-0.25	-0.35	-0.23	-0.08	-0.31	-0.13	0.02	*n*	*n*	*n*
History of GTC seizures	-0.27<br>*	-0.19	-0.36<br>h **	-0.11	-0.11	-0.11	-0.14	-0.13	-0.08	-0.11	-0.12	-0.05	-0.07	-0.09	0.00	-0.08	-0.11	0.04	0.01	-0.03	0.17

	**Pre-operative trajectory – relative rate of change**			**Performance at pre-operative assessment**			**Performance at first post-operative assessment**			**Performance at last post-operative assessment**			**Change from pre- to first post-operative assessment**			**Change from pre- to last post-operative assessment**			**Post-operative trajectory – relative rate of change**		
	**FSIQ**	**VIQ**	**PIQ**	**FSIQ**	**VIQ**	**PIQ**	**FSIQ**	**VIQ**	**PIQ**	**FSIQ**	**VIQ**	**PIQ**	**FSIQ**	**VIQ**	**PIQ**	**FSIQ**	**VIQ**	**PIQ**	**FSIQ**	**VIQ**	**PIQ**
Three or more seizure types at time of pre-operative evaluation (versus two or fewer seizure types)	0.06	0.02	-0.03	-0.26 *	-0.18 *	-0.22 **	-0.27	-0.16	-0.13	-0.20	-0.13	-0.08	-0.09	-0.14	-0.05	-0.06	-0.19	-0.01	-0.38	-0.07	*n*
Number of ASMs at pre-operative assessment	-0.14	-0.19 *	-0.11	-0.26 *h* ***	-0.19 *h* ***	-0.28 *h* ***	-0.35 *h* ***	-0.27 *h* ***	-0.33 *h* ***	-0.36 *h* ***	-0.29 *h* ***	-0.32 *h* ***	-0.12	-0.12	-0.19 *h* **	-0.15	-0.12 *	-0.16 **	-0.23	-0.01	0.08
Total number of ASMs trialled from epilepsy onset to pre-operative evaluation	0.02	-0.05	-0.15	-0.35 *h* ***	-0.29 *h* ***	-0.36 *h* ***	-0.36 *h* ***	-0.28 *h* ***	-0.37 *h* ***	-0.35 *h* ***	-0.28 *h* ***	-0.33 *h* ***	-0.03	-0.04	-0.15 *	0.00	-0.04	-0.09	-0.05	0.07	0.17
Bilateral MRI abnormalities (versus unilateral MRI abnormalities)	-0.06	-0.19	-0.06	-0.21	-0.15	-0.18 *	-0.28 *	-0.16	-0.18	-0.35 *	-0.21	-0.24 *	-0.11	-0.23	-0.14	-0.17	-0.31 *	-0.24	*n*	*n*	*n*
Focal MRI abnormalities (versus Diffuse/Multifocal/Negative)	0.14	0.11	0.19	0.38 *h* ***	0.27 *h* ***	0.34 *h* ***	0.42 *h* ***	0.31 *h* ***	0.42 *h* ***	0.45 *h* ***	0.33 *h* ***	0.44 *h* ***	0.03	0.08	0.13	0.08	0.11	0.15 *	0.17	0.03	0.02
Lesionectomy/ Lobectomy (versus Hemispherotomy/ Disconnection/ Corpus callosotomy)	-	-	-	-	-	-	0.52 *h* ***	0.40 *h* ***	0.55 *h* ***	0.53 *h* ***	0.42 *h* ***	0.59 *h* ***	0.02	0.02	0.19 *	0.13	0.06	0.21 *	0.38	0.37 *	0.51 *h* ***
Right-sided surgery (versus Left-sided surgery)	-	-	-	-	-	-	-0.04	0.05	-0.12	-0.10	0.01	-0.20 *h* **	0.00	-0.03	0.00	-0.11	-0.09	-0.07	-0.14	-0.19	-0.19

	**Pre-operative trajectory – relative rate of change**			**Performance at pre-operative assessment**			**Performance at first post-operative assessment**			**Performance at last post-operative assessment**			**Change from pre- to first post-operative assessment**			**Change from pre- to last post-operative assessment**			**Post-operative trajectory – relative rate of change**		
	**FSIQ**	**VIQ**	**PIQ**	**FSIQ**	**VIQ**	**PIQ**	**FSIQ**	**VIQ**	**PIQ**	**FSIQ**	**VIQ**	**PIQ**	**FSIQ**	**VIQ**	**PIQ**	**FSIQ**	**VIQ**	**PIQ**	**FSIQ**	**VIQ**	**PIQ**
Multilobar surgery (versus Unilobar surgery)	-	-	-	-	-	-	-0.19	-0.29 *	-0.33 *	-0.35 *	-0.36 ^{h} **	-0.38 ^{h} **	-0.02	-0.13	-0.01	-0.16	-0.21	-0.09	*n*	*n*	*n*
Aetiology: LEAT	-0.14	0.00	0.07	0.36 ^{h} ***	0.25 ^{h} ***	0.28 ^{h} ***	0.35 ^{h} ***	0.24 ^{h} **	0.31 ^{h} ***	0.35 ^{h} ***	0.26 ^{h} ***	0.27 ^{h} ***	0.06	0.16 *	0.14	0.03	0.15 *	0.04	0.02	0.11	-0.06
Aetiology: MTS	0.01	0.02	-0.01	-0.10	-0.15 *	-0.03	-0.03	-0.15	-0.07	0.04	-0.09	0.07	0.03	0.00	0.00	0.23 *	0.06	0.20 *	0.33 *	-0.05	0.14
Aetiology: FCD-II	0.29	0.28	0.02	-0.03	0.04	-0.01	-0.11	0.03	0.03	0.10	0.00	0.00	0.03	-0.05	0.09	-0.08	-0.04	0.06	*n*	0.22	*n*
Aetiology: Rasmussen encephalitis	*n*	-0.43 *	-0.37	-0.41 **	-0.28 *	-0.45 ^{h} **	-0.55 *	-0.32 *	-0.68 ^{h} ***	-0.58 **	-0.29 *	-0.66 ^{h} ***	-0.28	-0.01	-0.56 ^{h} ***	-0.35	0.12	-0.39 *	*n*	*n*	*n*
Seizure-free (versus Not seizure-free) [c]	-	-	-	-	-	-	0.37 ^{h} ***	0.24 ^{h} ***	0.27 ^{h} ***	0.47 ^{h} ***	0.32 ^{h} ***	0.34 ^{h} ***	0.12	0.09	0.13	0.33 ^{h} ***	0.24 ^{h} **	0.23 **	0.44 ^{h} **	0.36 ^{h} **	0.21
Off ASM (versus Receiving ASM/ Weaning ASM)	-	-	-	-	-	-	0.32 **	0.25 **	0.27 **	0.32 **	0.25 **	0.25 *	-0.12	-0.06	0.08	0.08	0.01	0.04	*n*	-0.06	-0.11

Associations between demographic, clinical and surgical variables and neuropsychological functioning were investigated using Pearson's correlation coefficient, Spearman's rank correlation coefficient, and rank-biserial correlation coefficient, as appropriate. Age of epilepsy onset, age at surgery, and duration of epilepsy were analysed separately, using partial correlations, to take into account the intercorrelated nature between these clinical characteristics.

Abbreviations: ASM = antiseizure medication; FCD-II = focal cortical dysplasia type II; FSIQ = Full-Scale IQ; GTC = generalised tonic-clonic; LEAT = low-grade epilepsy-associated tumour; MTS = mesial temporal sclerosis; PIQ = Performance IQ; SEN = special educational needs; VIQ = Verbal IQ.

p-values were obtained prior to correction for multiple comparison using the Holm method, which was then applied sequentially to each column. ^{h} Denotes if the p-value survived correction for multiple comparison.

n = analysis not performed due to the comparison group having a sample size smaller than $N = 10$.

[a] For preoperative trajectory, this was the first assessment patients had had. For postoperative performance, this was the preoperative assessment closest to surgery (the last preoperative assessment). For postoperative trajectory, this was the postoperative performance closest to surgery (first postoperative assessment).

[b] For preoperative neuropsychology assessment, duration of epilepsy was calculated from time of epilepsy onset to time of neuropsychology assessment. For postoperative neuropsychology assessment, duration of epilepsy was calculated from time of epilepsy onset to time of surgery.

[c] For postoperative assessment, this was seizure outcome at one-year follow-up. For postoperative trajectories, this was seizure outcome at last follow-up.

*p <0.05

**p <0.01

***p <0.001

Supplementary Table 15. Neuropsychological functioning at first postoperative assessment in seizure-free patients not receiving ASMs versus receiving/weaning ASMs.

	**Off ASM**	**On ASM/Weaning**	
	**Score**, Md [IQR] (range)	**Score**, Md [IQR] (range)	**p**
**IQ domains**			
**FSIQ** *N* = 148 (Off ASM = 27; On ASM/Weaning = 121)	87 [74, 100] (49, 130)	78 [66, 91] (43, 122)	0.057
**Verbal IQ** *N* =225 (Off ASM = 42; On ASM/Weaning = 183)	91 [71, 98] (50, 130)	80 [67, 96] (45, 126)	0.068
**Performance IQ** *N* = 205 (Off ASM = 35; On ASM/Weaning = 170)	93 [82, 105] (50, 127)	86 [72, 100] (40, 133)	0.051
**Working Memory** *N* = 183 (Off ASM = 36; On ASM/Weaning = 147)	91 [78, 102] (56, 124)	83 [68, 97] (50, 136)	0.026 *
**Processing Speed** *N* = 177 (Off ASM = 34; On ASM/Weaning = 143)	88 [78, 102] (50, 123)	80 [68, 91] (49, 121)	0.012 *
**Academic attainment**			
**Reading** *N* = 191 (Off ASM = 30; On ASM/Weaning = 161)	93 [75, 103] (45, 121)	85 [69, 97] (40, 129)	0.158
**Spelling** *N* = 178 (Off ASM = 29; On ASM/Weaning = 149)	90 [76, 105] (54, 118)	86 [73, 98] (40, 128)	0.181
**Numeracy** *N* = 178 (Off ASM = 29; On ASM/Weaning = 149)	94 [75, 106] (56, 134)	81 [65, 93] (40, 139)	0.028 *

Abbreviations: ASM = antiseizure medication; FSIQ = Full-Scale IQ.

*p <0.05

**p <0.01

***p <0.001

Supplementary Table 16. Postoperative neuropsychological trajectories in seizure-free patients who were no longer receiving ASMs versus receiving or weaning ASMs.

	**Off ASM**	**On ASM/Weaning**	**p**
**IQ domains**			
**FSIQ**<br>*N* = 47 (Off ASM = 30; On ASM/Weaning = 17)	1.8 [0.3, 2.2]<br>(-1.1, 8.6)	1.2 [-0.6, 2.2]<br>(-2.4, 3.5)	0.211
**Verbal IQ**<br>*N* = 63 (Off ASM = 39; On ASM/Weaning = 24)	1.3 [0.0, 3.1]<br>(-2.7, 7.0)	-0.1 [-1.4, 2.0]<br>(-9.6, 5.4)	0.026<br>*
**Performance IQ**<br>*N* = 65 (Off ASM = 40; On ASM/Weaning = 25)	1.3 [-0.2, 2.7]<br>(-2.0, 10.6)	0.0 [-2.0, 2.6]<br>(-6.1, 6.1)	0.205
**Working Memory**<br>*N* = 51 (Off ASM = 30; On ASM/Weaning = 21)	1.4 [0.7, 2.9]<br>(-10.4, 9.0)	0.0 [-1.8, 1.9]<br>(-4.1, 4.8)	0.032<br>*
**Processing Speed**<br>*N* = 50 (Off ASM = 28; On ASM/Weaning = 22)	0.7 [-0.1, 2.3]<br>(-4.3, 10.0)	0.0 [-1.7, 2.0]<br>(-5.5, 6.4)	0.214
**Academic attainment**			
**Reading**<br>*N* = 50 (Off ASM = 28; On ASM/Weaning = 22)	0.9 [-0.2, 2.2]<br>(-1.1, 10.5)	-0.7 [-2.5, 1.9]<br>(-6.8, 4.7)	0.034<br>*
**Spelling**<br>*N* = 52 (Off ASM = 32; On ASM/Weaning = 20)	1.0 [-0.1, 2.4]<br>(-2.1, 7.8)	-0.3 [-1.7, 1.5]<br>(-5.7, 5.0)	0.026<br>*
**Numeracy**<br>*N* = 53 (Off ASM = 32; On ASM/Weaning = 21)	-1.1 [-1.9, 0.7]<br>(-8.3, 5.6)	-2.6 [-4.1, 0.4]<br>(-19.0, 7.5)	0.200

Abbreviations: ASM = antiseizure medication; FSIQ = Full-Scale IQ.

*p <0.05

**p <0.01

***p <0.001
